# High throughput discovery of influenza virus neutralizing antibodies from phage-displayed synthetic antibody libraries

**DOI:** 10.1038/s41598-017-14823-w

**Published:** 2017-10-31

**Authors:** Ing-Chien Chen, Yi-Kai Chiu, Chung-Ming Yu, Cheng-Chung Lee, Chao-Ping Tung, Yueh-Liang Tsou, Yi-Jen Huang, Chia-Lung Lin, Hong-Sen Chen, Andrew H.-J. Wang, An-Suei Yang

**Affiliations:** 1 Genomics Research Center, Academia Sinica, 115 Taipei, Taiwan; 2 Institute of Biological Chemistry, Academia Sinica, 115 Taipei, Taiwan

## Abstract

Pandemic and epidemic outbreaks of influenza A virus (IAV) infection pose severe challenges to human society. Passive immunotherapy with recombinant neutralizing antibodies can potentially mitigate the threats of IAV infection. With a high throughput neutralizing antibody discovery platform, we produced artificial anti-hemagglutinin (HA) IAV-neutralizing IgGs from phage-displayed synthetic scFv libraries without necessitating prior memory of antibody-antigen interactions or relying on affinity maturation essential for *in vivo* immune systems to generate highly specific neutralizing antibodies. At least two thirds of the epitope groups of the artificial anti-HA antibodies resemble those of natural protective anti-HA antibodies, providing alternatives to neutralizing antibodies from natural antibody repertoires. With continuing advancement in designing and constructing synthetic scFv libraries, this technological platform is useful in mitigating not only the threats of IAV pandemics but also those from other newly emerging viral infections.

## Introduction

Seasonal and pandemic influenza virus infections lead to substantial social and economic burden worldwide^1,2^. According to World Health Organization (WHO), seasonal influenza virus infections reach around 1 billion cases each year, with 3–5 million annual cases of severe illness and 250,000–500,000 annual deaths, who are mostly high risk groups such as elderly, infants, and people with underlying chronical illness^3^. Pandemic influenza A virus (IAV) outbreaks pose much more severe challenges; three major influenza pandemics occurred in the past century: 1918 H1N1 Spanish, 1957 H2N2 Asian, and 1968 H3N2 Hong Kong pandemic^4^, leading to total deaths of about 50~100 million worldwide. The 21th century’s first pandemic, unpredictedly caused by 2009 H1N1 influenza virus (Mexico) of swine origin, was reported by WHO of millions of cases and 16813 deaths (as of 2010/3/19)^4^. While the next pandemic influenza outbreak has been unpredictable with our current knowledge, the concern over an avian strain of pandemic virus is certainly warranted, as revealed by the emergence of avian influenza strains, including H5N1 (Hong Kong) in 1997, H7N9 (China), H10N8 (China) and H6N1 (Taiwan) in 2013 and H5N6 (Hong Kong) in 2014^5,6^, among which the highly pathogenic avian influenza H5N1 (Hong Kong) and H7N9 (China) are of particular concern^7–12^. If virus genome reassortment or antigenic drift were to result in the new H7N9/H5N1 avian influenza virus transmissible among humans, a new pandemic could arise.

Counter measures for IAV epidemics and pandemics have not provided enough protection to human population. Seasonal trivalent or quardrivalent influenza vaccines are conditionally effective, depending on the match of the predicted vaccine strains and the strains in circulation each year^13,14^. More importantly, the majority of human population does not have protective immunity against pandemic influenza viruses and vaccines against the pandemic strains would be available with considerable delay of at least 5~6 months after the availability of the vaccine virus to vaccine manufacturers^14,15^. By the time when the vaccine becomes available, substantial mortality, morbidity and economic loss could have already occurred. Universal vaccines with efficacy against a broad spectrum of influenza strains are still under development^1,13,16^; even effective vaccines are available, there would be a delay of about 2 weeks before developing protective immunity^14,15^. Stockpile of antiviral drugs (mostly neuraminidase inhibitors such as oseltamivir (Tamiflu)) as strategy for curbing influenza pandemics has been questioned^17^. Moreover, influenza strains with pandemic threats could be resistant to current antivirals, as drug-resistant influenza virus strains have occurred due to antigenic shift and antigenic drift^3,18^.

Passive immunotherapy with neutralizing antibodies to treat severe IAV infections can be a viable strategy in mitigating the threats of influenza epidemic and pandemic outbreaks^15,19–27^. Human antibody discoveries based on culturing/cloning human single memory and plasmablast B cells and displaying human antibody repertoires on phage particles^21^ have resulted in potent human neutralizing antibodies^3,22,23^, many of which are broadly neutralizing antibodies (bnAbs) and are being tested for passive immunotherapy in human trials^15^. However, affinity matured human antibodies with neutralizing efficacy against pandemic strains harboring genetic makeup unknown to human immune systems could be difficult to attain^21^. Moreover, neutralization escape variants could eventually emerge due to the selection pressure of the widespread treatments with the neutralizing antibodies^26,28^. There are increasingly urgent needs to rapidly develop neutralizing antibodies to respond to immediate threats of the emerging viral infections. In this work, using the 2009 pandemic IAV H1N1 A/California/07/2009 (H1N1 CA/09 in short) as a model system, we demonstrate that neutralizing antibodies against pandemic IAV hemagglutinin (HA) can be rapidly attained from phage-displayed synthetic antibody libraries, which are designed with antibody bioinformatics and constructed with recombinant methodologies^29^. The phage-displayed synthetic antibody libraries enable a high throughput antibody discovery platform to attain functional antibodies with neutralizing capability against influenza virus infection, providing antibody candidates for passive immunotherapies that could contribute mitigating the threats of influenza pandemic outbreaks. The high throughput discovery of neutralizing antibodies can be completed in about 4 weeks once the recombinant antigens become available; the same technology could also provide antibody-based counter measures against threats from other newly emerging viral infections.

## Results

### Phage-displayed synthetic antibody libraries enable high throughput discovery of anti-IAV neutralizing antibodies

**Figure 1:**
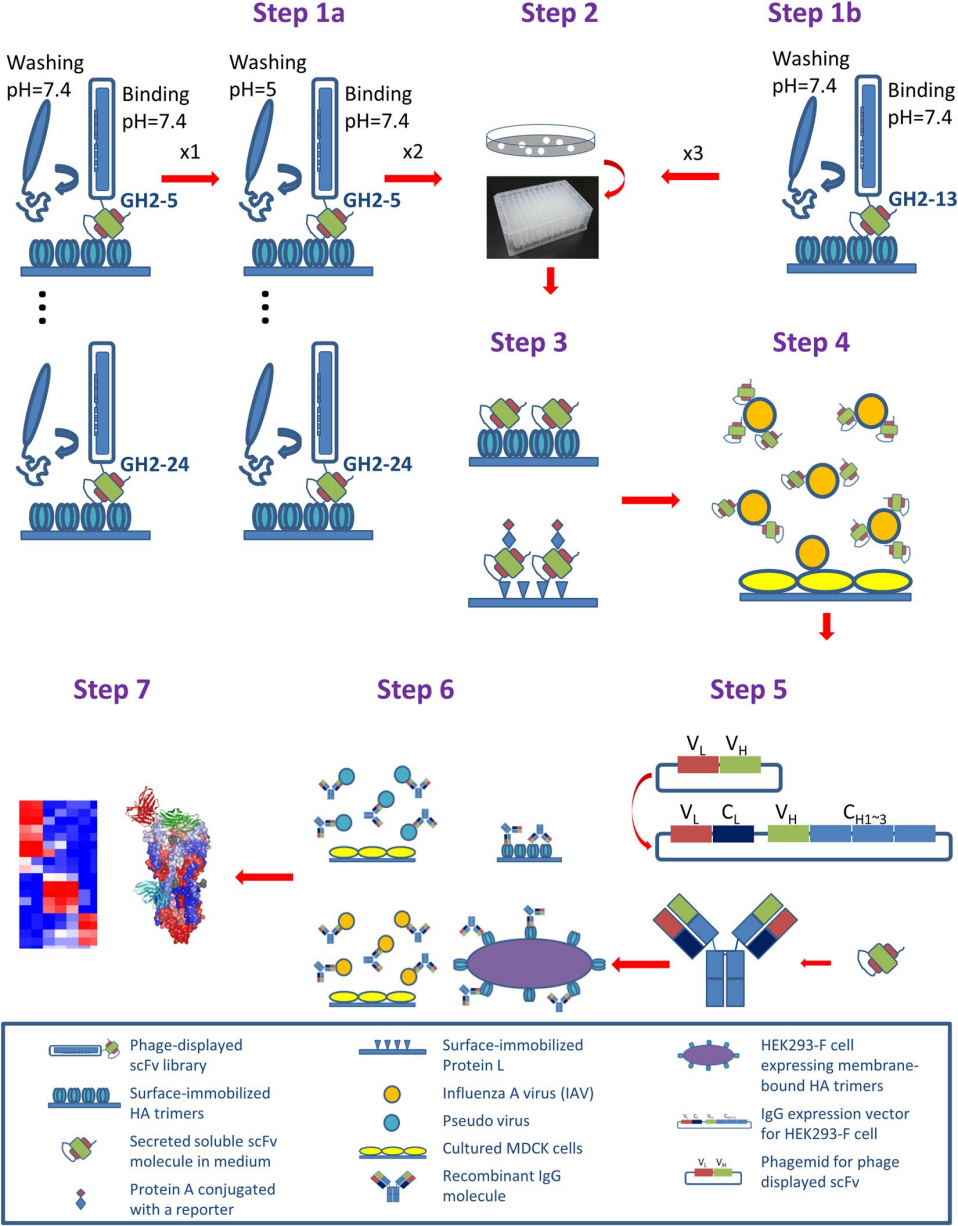
Schematic follow chart for the high throughput discovery of anti-IAV neutralizing antibodies from the phage-displayed synthetic antibody libraries. Step 1a: Parallel biopanning of anti-HA scFvs from 15 phage-displayed synthetic scFv libraries (GH2-5~24) under different pH conditions (results shown in Fig. 2). Step1b: Biopanning of anti-HA scFvs from the GH2-13 phage-displayed scFv library at neutral pH (results shown in Fig. 2). Step 2: Picking single colonies for soluble monoclonal scFvs secreted from cultured *E. coli* cells in 96-well deep well plate. Step 3: Screening for functional scFvs binding to HA trimer, Protein A, and Protein L with ELISA (results shown in Fig. 3). Step 4: Screening the functional scFvs for IAV neutralization with micro-neutralization assay (results shown in Fig. 3). Step 5: Reformating the IAV neutralizing functional scFvs to human IgG1s, expressed with 293-F cells. Step 6: Assessing the EC_50_’s (binding assays with ELISA and cell flow cytometry) and IC_50_’s (micro-neutralization assays with IAV and pseudo virus) of the recombinant IgGs (results shown in Figs 4~7). Step 7: Grouping epitopes with competition ELISA and epitope mapping with structure determination (results shown in Figs 8~9). Technical details are shown in Methods.

Success of high throughput discovery of anti-IAV neutralizing antibodies hinges on the contents of the antibody libraries. Antibody discovery based on the M13 phage-displayed scFv libraries has been one of the most successful methodologies with high throughput capability^30^. To fully exploit the advantages of the phage display methodology, we have developed phage-displayed synthetic scFv libraries, for which the scFv variants are stably folded with the CDR sequences encoding amino acid residues with high propensities for protein recognitions^29^. The foldable scFv variants had been pre-selected during the library construction with Protein A and Protein L binding to ensure the foldability of the scFv molecules expressed on the phage surface as fusion protein or in soluble form independent of the phage system^29^. Such prerequisite embedded in the library construction procedure has resulted in highly functional synthetic scFv libraries suitable for high throughput screening of the soluble scFv variants after the scFv variant population being reduced by several rounds of biopanning (Fig. 1); most of the synthetic scFv variants can be prepared as soluble scFv molecules with more than 1 µg/mL (or 33 nM) in crude *E. coli* culture in 96-well deep well plate overnight, feasible for many high throughput functional assays^31^. By contrast to the unpredictable expression of the scFv molecules from conventional phage-displayed scFv libraries constructed from natural antibody gene repertories, where the scFv molecules are frequently unable to be expressed in soluble form with sufficient concentration, the stable expression of functional synthetic scFvs enables a high throughput screening procedure effectively compatible with the high throughput micro-neutralization assay used in screening neutralizing antibodies against IAV infection^32^. The overall schematic high throughput procedure enabled by the phage-displayed synthetic antibody libraries is summarized in Fig. 1.

To enhance the coverage of the phage-displayed synthetic antibody libraries, we constructed 15 phage-displayed single-framework synthetic antibody libraries (Step 1a in Fig. 1). The framework of the synthetic antibody libraries is based on the human IGKV1-NL1*01/IGHV3-23*04 germline sequence^29^; the CDR-L1~H2 sequence constructs for the 15 synthetic scFv libraries are detailed in Supplementary Table S1, for which the protein antigen-recognition capabilities have been demonstrated previously^29^. The CDR-H3 constructs for each of the 15 synthetic antibody libraries (GH2-5~24) with CDR-H3 sequence length ranging from 5 to 24 residues are listed in Supplementary Table S2. Each of the libraries has been constructed with complexity of more than 10^9^; the diversity qualities of the libraries have been validated with Illumina Mi-seq high throughput sequencing (Supplementary Figure S1), indicating that the amino acid type distributions on the CDR residue positions of the GH2-5~24 libraries have been constructed according to the CDR designs shown in Supplementary Tables S1~S2.

### Anti-IAV monoclonal scFvs are selected and screened from the phage-displayed synthetic antibody libraries on high throughput platform

**Figure 2:**
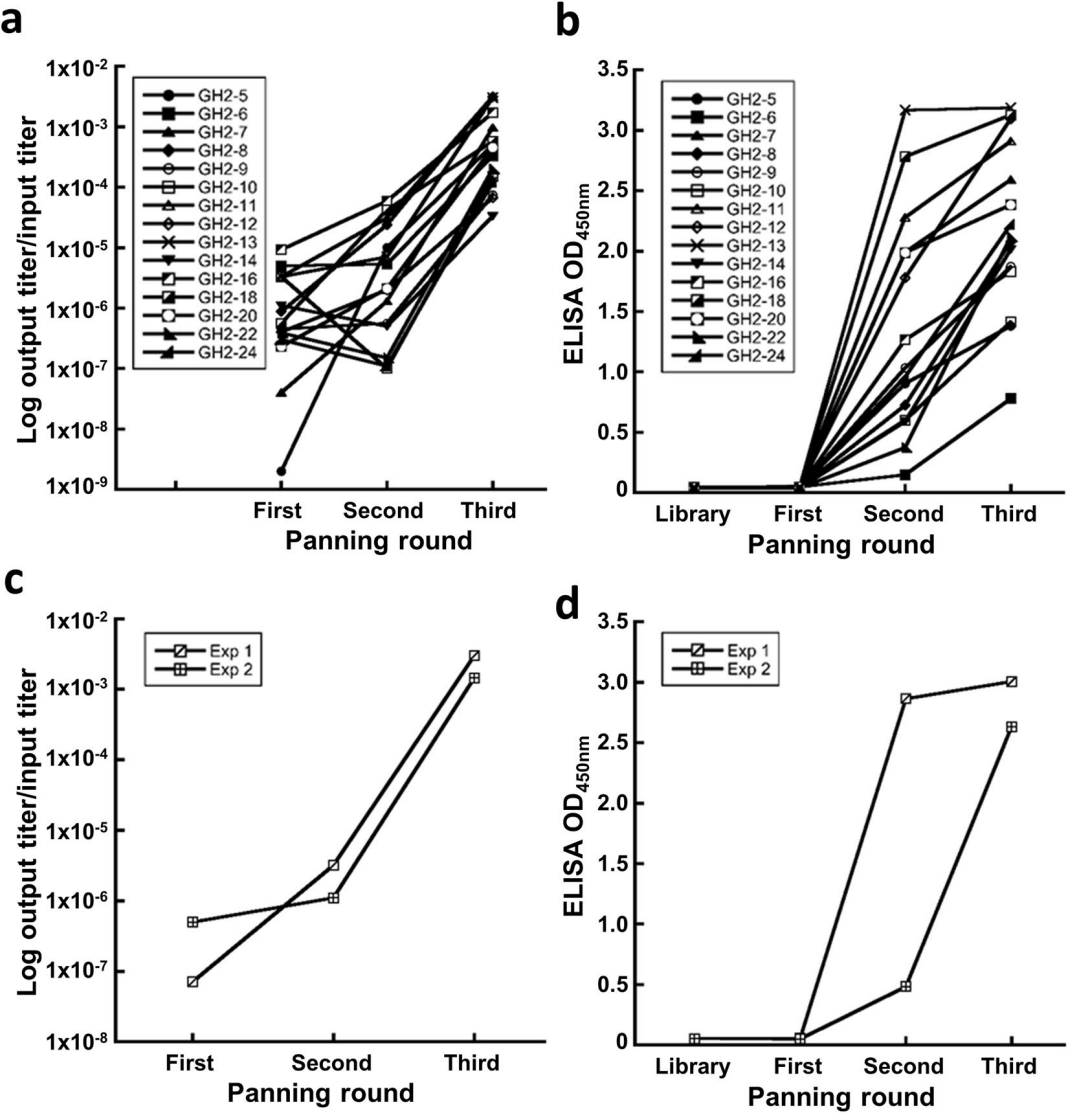
Results of biopanning of the synthetic antibody libraries against immobilized HA trimer of H1N1 CA/09. (**a**) 15 GH2 phage-displayed scFv libraries were selected for HA binding. The y-axis shows the ratio of the output/input titer of the phage library in each of the biopanning rounds (x-axis). (**b**) The polyclonal soluble scFvs secreted in the *E. coli* culture media of the output phage libraries were assayed for HA trimer binding with ELISA (y-axis) for the biopanning rounds (x-axis) with each of the 15 phage-displayed scFv libraries. (**c**)~(**d**) The descriptions are the same as in (**a**) and (**b**) respectively for the biopanning protocol of the Step 1b in Fig. 1. The experiments were repeated twice, as shown in the panels. Experimental details are described in Methods.

Anti-IAV antibodies were attained from the 15 phage-displayed synthetic scFv libraries through three rounds of biopanning with different pH in washing condition (Step 1a and Step 1b in Fig. 1). The binding phase of the biopanning, where the phage-displayed scFvs bind to immobilized HA trimers of the 2009 pandemic IAV strain H1N1 CA/09, was always carried out at neutral pH (7.4); the pH condition of the washing phase, where the unbound phage particles were removed from the antigen surface, was neutral for the first round, which was then followed by two rounds of biopanning with washing phase of pH = 5 (Step 1a in Fig. 1). The pH conditions mimic the pH change during the endocytosis of the host receptor-bound IAV from neutral environment to mildly acidic condition in the late endosome, where the conformational change of the HA trimer induced by the exposure to the acidic condition results in the infection of the host cell through the IAV membrane fusion with that of the host cell^33^ – surviving the acidic washing phase is a necessary condition for the scFv to neutralize IAV infection as a fusion inhibitor. For comparison, a control experiment with neutral binding and washing phase in all three rounds of biopanning (Step 1b in Fig. 1) was compared with one of the scFv libraries (GH2-13) in Step 1a, so as to contrast the effects of the acidic washing phase in the biopanning procedure of Step 1a. Both biopanning protocols were effective in resulting in HA binding scFvs after two to three rounds of biopanning (Fig. 2); the biopanning results indicate that all the 15 scFv libraries contain HA trimer-binding scFv variants, for which the soluble scFv molecules in the *E. coli* culture media are capable of binding to the surface-immobilized HA trimer.

**Figure 3:**
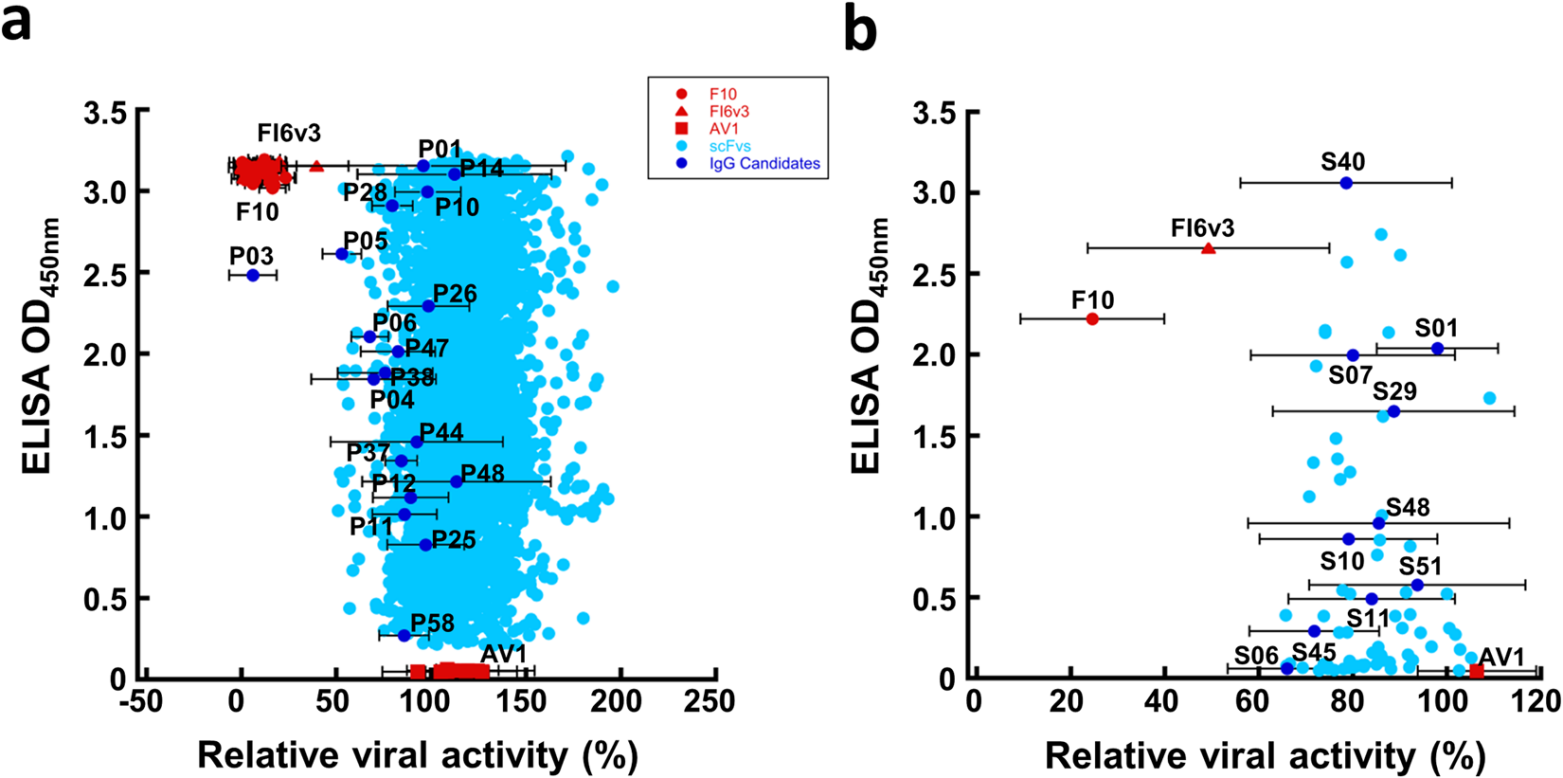
High throughput screening of anti-IAV H1N1 CA/09 neutralizing scFvs binding to H1N1 CA/09 HA trimer. (**a**) High throughput screening results (Step 3~4 in Fig. 1) for more than 5000 monoclonal scFvs with the protocol of Step 1a in Fig. 1 are summarized in this panel. The y-axis shows the ELISA signal of the monoclonal scFvs binding to H1N1 CA/09 HA trimer; the x-axis shows the relative viral activity based on the viral activity ratio measured in the microneutralization assay with IAV H1N1 CA/09 after and before addition of the scFv culture medium to the assay system – 100% relative viral active means no inhibition of the scFv to the IAV infection to the MDCK cell; 0% relative viral activity means complete inhibition of the infection. Positive control scFvs derived from F10 and FI6v3 IgGs show almost 0% relative viral activity in the assay and negative control scFv derived from AV1 IgG shows almost 100% relative viral activity. The dark blue data points labelled with antibody names are selected for IgG reformatting (Supplementary Tables S3~S4). The error bars associated with these data points were determined with at least three repeats of the microneutralization measurements. (**b**) The description is the same as in panel (a) for the high throughput screening results (Step 3~4 in Fig. 1) for more than 100 monoclonal scFvs with the protocol of Step 1b in Fig. 1 are summarized in this panel. Experimental details are described in Methods.

Monoclonal scFvs were screened in high throughput format for folding into stable variable domain antibody fragment (scFv) (Step 3 in Fig. 1), binding to immobilized HA trimer (Step 3 in Fig. 1), and neutralizing IAV infection with micro-neutralization assay (Step 4 in Fig. 1). The monoclonal scFvs were expressed in *E. coli* culture media in 96-well deep well plate (Step 2 in Fig. 1), and the overnight culture supernatants without further scFv purification were ready for high throughput assays. One unique feature of the single-framework scFv variants from the synthetic GH2-5~24 scFv libraries is that the scFv molecule binds to Protein A and Protein L simultaneously in solution^31^, and hence a sandwich ELISA with immobilized Protein L and reporter-conjugated Protein A can be used to identify well-folded scFv molecules (Step 3 in Fig. 1). Only the well-folded scFvs (more than 90% of the selected clones in Step 2) were assayed for HA-binding and IAV-neutralization (Steps 3~4 in Fig. 1). The HA-binding signals of more than 5000 monoclonal scFvs with properly folded structures selected from all 15 scFv libraries are plotted against their IAV-neutralization potencies in Fig. 3a. In comparison, the corresponding plot for the control experiment starting from Step 1b is shown in Fig. 3b. The comparison indicates that the acidic condition in the washing phase had improved the IAV-neutralization potencies of the selected scFvs by a small margin. 28 sequence-wise non-redundant scFvs with IAV-neutralization potencies (data points marked in Fig. 3a,b) from both set of scFv variants were selected for IgG1 reformatting (Step 5 in Fig. 1). The CDR sequences of these IgG1s are summarized in Supplementary Table S3. The expression yields for these IgG1s are shown in Supplementary Table S4; the SDS-PAGE of these IgG1s are shown in Supplementary Figure S3.

### Anti-IAV neutralizing IgG antibodies are reformatted from selected monoclonal scFvs

**Figure 4:**
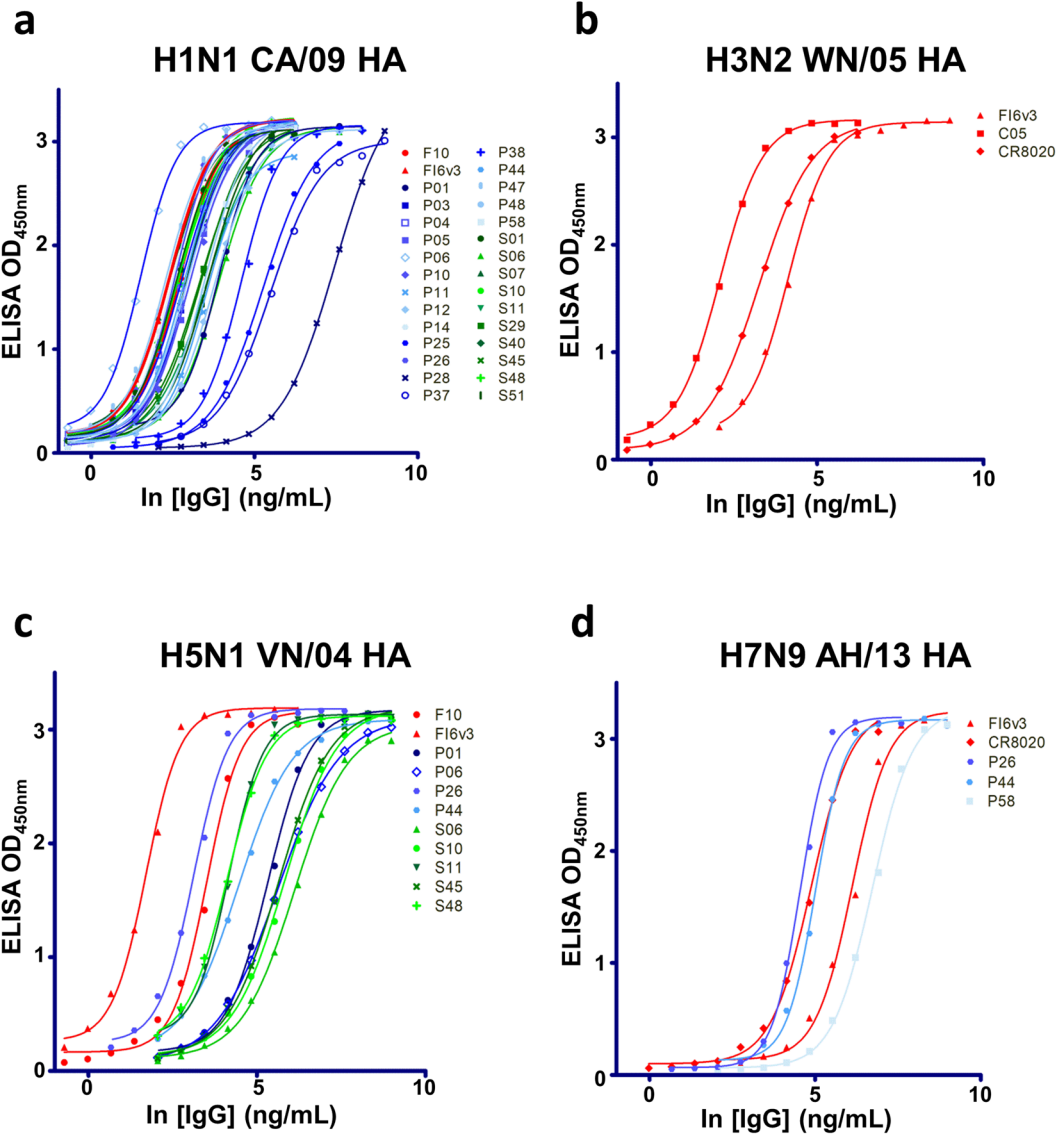
EC_50_ measurements with ELISA for the IgGs reformatted from the selected anti-HA trimer scFvs. (**a**)~(**d**) The IgGs were reformatted from the selected anti-HA trimer scFvs as shown in Fig. 3. The experimental details are described in Methods. The CDR sequences of these IgGs are shown in Supplementary Table S3, and the numerical values of the EC_50_’s and the sigmoidal curve fitting correlation coefficients are listed in Supplementary Table S4. The positive control IgGs: F10^7^, FI6v3^34^, C05^41^, and CR8020^42^ were prepared as described in Methods.

Antigen (HA trimer) binding affinity (50% maximal effective concentration – EC_50_) and IAV-neutralization (50% maximal inhibitory concentration – IC_50_) measurements (Step 6 in Fig. 1) for the 28 IgGs led to the confirmation of IAV-neutralizing IgGs with antigen-binding affinity approaching the affinity ceiling of affinity-matured anti-IAV antibodies derived from *in-vivo* immune systems (Fig. 4a). Since the IgGs were selected and screened against the HA trimer of the strain H1N1 CA/09, the EC_50_’s of these IgGs are approaching the affinity ceiling for the H1N1 CA/09 HA trimer of the control positive antibodies (F10 and FI6v3) reported previously^7,34^ (Fig. 4a). Some of these IgGs are able to cross-react with the HA trimer of another group 1 subtype IAV H5N1 VN/04, albeit with lower affinities (Fig. 4c). By contrast, none of the 28 IgGs cross-react with the HA trimers of group 2 subtype H3N2 WN/05 (Fig. 4b), and only three IgGs are able to cross-react with the HA trimer of group 2 subtype H7N9 AH/13 (Fig. 4d). The EC_50_’s are summarized in Supplementary Table S4.

**Figure 5:**
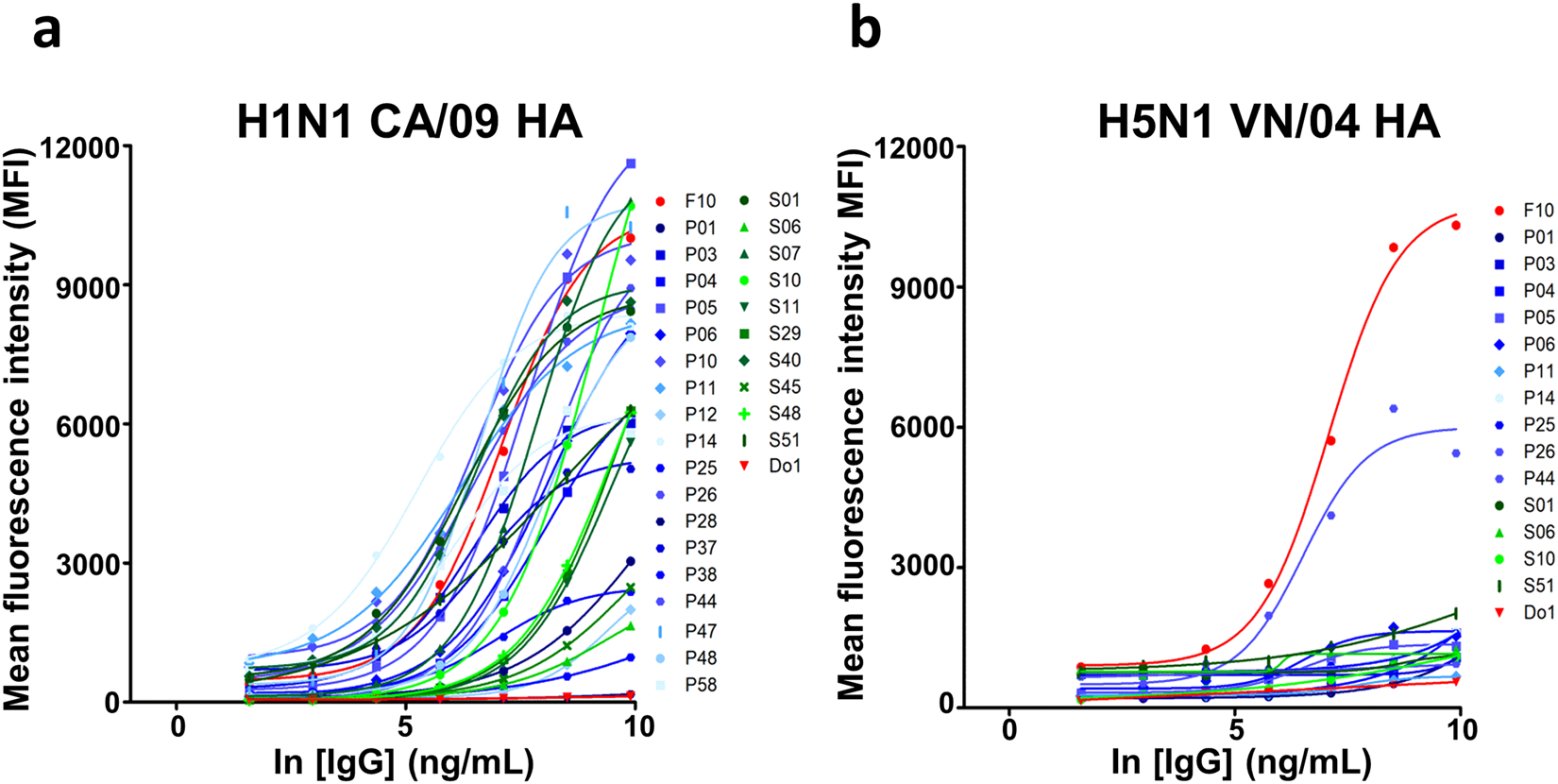
Flow cytometry-based EC_50_ measurements with mean fluorescence intensity (MFI) for the IgGs binding to cell surface-expressed H1N1 CA/09 and H5N1 VN/04 HA trimers on 293 T cells. (**a**),(**b**) The IgGs were reformatted from the selected anti-HA trimer scFvs as shown in Fig. 3. The experimental details are described in Methods. The CDR sequences of these IgGs are shown in Supplementary Table S3, and the numerical values of the EC_50_’s and the maximal MFI are listed in Supplementary Table S4. The experimental details are described in Methods.

**Figure 6:**
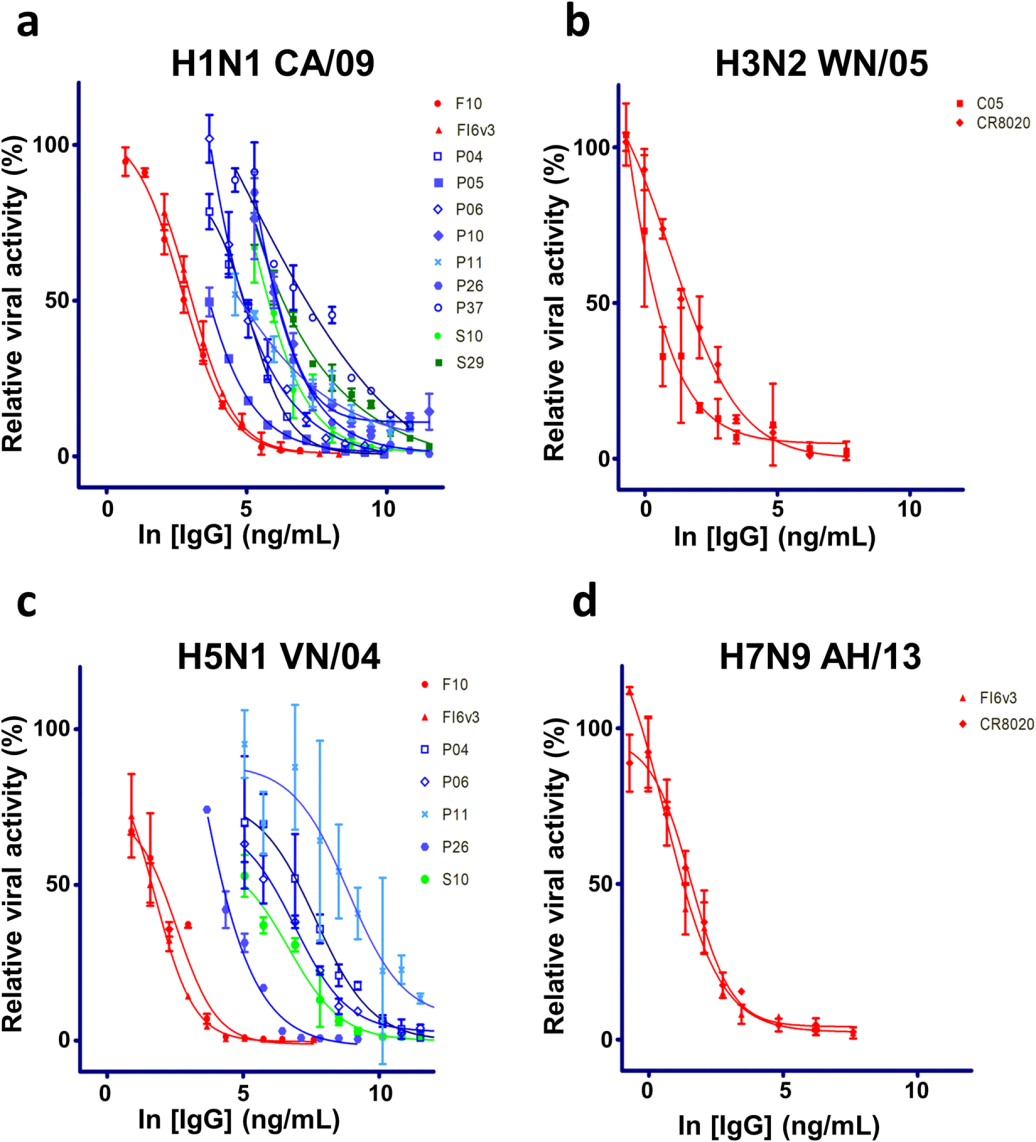
IC_50_ measurements with pseudo virus-based microneutralization assay for the IgGs reformatted from the selected anti-HA trimer scFvs. (**a**)~(**d**) The IgGs were reformatted from the selected anti-HA trimer scFvs as shown in Fig. 3. The y-axis shows the relative viral activity plotted against the IgG concentration (x-axis). The experimental details are described in Methods. The CDR sequences of these IgGs are shown in Supplementary Table S3, and the numerical values of the IC_50_’s are listed in Supplementary Table S4. The error bars associated with the data points are calculated with at least three independent repeats of the microneutralization assay. The positive control IgGs: F10^7^, FI6v3^34^, C05^41^, and CR8020^42^ were prepared as described in Methods.

To address the biological relevance of the ELISA-based EC_50_’s, we determined MFI-based EC_50_’s with cell flow cytometry measuring the IgG-HA trimer binding on 293 T cells over-expressing the HA trimers on the cell surfaces (Fig. 5 and Supplementary Table S4). Qualitative, all the IgGs binding to ELISA surface-immobilized H1N1 CA/09 HA trimer also bind to 293 T surface expressed H1N1 CA/09 HA trimer (Figs 4a, 5a), indicating that immobilization of HA trimers on solid ELISA surface does not substantially change the biologically relevant structure of the HA trimer. As expected, the IgGs are also able to cross-react with the cell surface-expressed H5N1 VN/05 HA trimer, but with significant reduction of the antibody-HA affinities (Fig. 5b). Also as expected, none of the IgGs cross-react with the cell surface-expressed H3N2 WN/05 HA trimer. We were not able to express the H7N9 AH/13 HA trimer on 293 T cell surface and hence the few IgGs cross-reacting with the H7N9 AH/13 HA trimer based on the ELISA measurements in Fig. 4d cannot be confirmed in the cell-based binding experiments. Nevertheless, pseudo virus-based micro-neutralization assays indicate that these potentially cross-reacting IgGs cannot neutralize the H7N9 AH/13 IAV infection (Fig. 6 and Supplementary Table S4).

Pseudo virus-based micro-neutralization assays indicate that about a quarter of the IgGs binding the H1N1 CA/09 HA trimer can neutralize the pseudo virus infection to the extent, for which the IC_50_’s are measurable in the micro-neutralization assay (Fig. 6a and Supplementary Table S4), and some of the IgGs cross-reacting with the H5N1 VN/04 HA trimer are also neutralizing antibodies against the H5N1 VN/04 pseudo virus (Fig. 6c and Supplementary Table S4). As expected from the binding assays, these IgGs do not have neutralizing effect against the group 2 pseudo viruses (Fig. 6b,d). The results of the pseudo virus-based micro-neutralization assays for the non-neutralizing IgGs are shown in Supplementary Figure S2.

**Figure 7:**
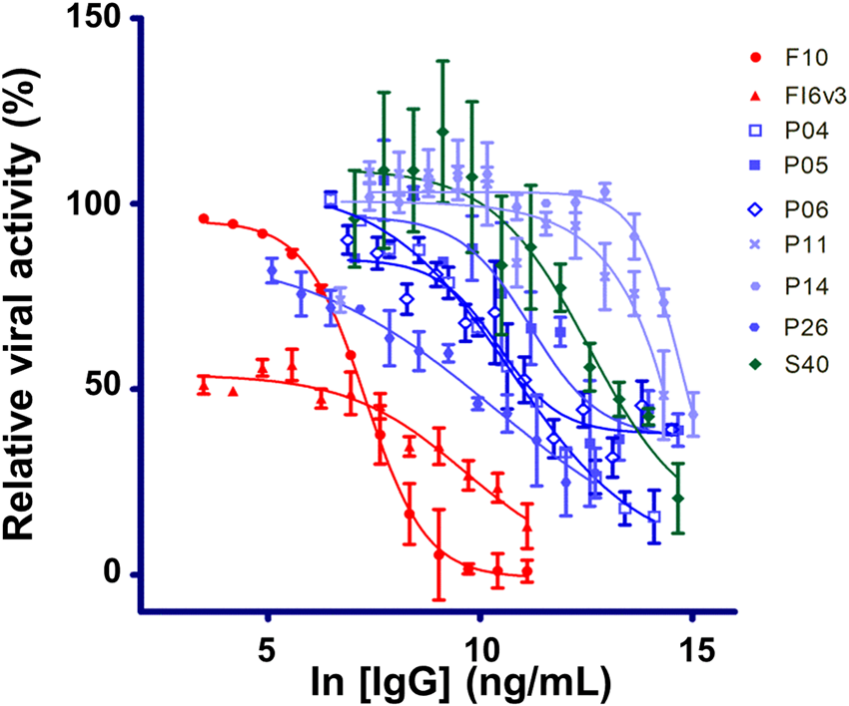
IC_50_ measurements with IAV H1N1 CA/09 microneutralization assay for the IgGs reformatted from the selected anti-HA trimer scFvs. The IgGs were reformatted from the selected anti-HA trimer scFvs as shown in Fig. 3. The y-axis shows the relative viral activity plotted against the IgG concentration (x-axis). The experimental details are described in Methods. The CDR sequences of these IgGs are shown in Supplementary Table S3, and the numerical values of the IC_50_’s are listed in Supplementary Table S4. The error bars associated with the data points are calculated with at least three independent repeats of the microneutralization assay. The positive control IgGs: F10^7^ and FI6v3^34^ were prepared as described in Methods.

To address the biological relevance of the pseudo virus-based micro neutralization assays, we determined the IC_50_’s of the IgGs with IAV strain H1N1 CA/09 and compared the IC_50_’s measured by the two micro-neutralization assays. Only a subset of the neutralizing IgGs identified by the pseudo virus-based micro-neutralization assays can neutralize the real virus infection to the extent, for which the IC_50_’s are measurable in the micro-neutralization assay (Fig. 7 and Supplementary Table S4), indicating that the pseudo virus-based micro-neutralization assay is more sensitive in comparison with the micro-neutralization assay with the real virus. Overall, only the IgGs identified with the protocol of the Step 1a are neutralizing antibodies, indicating that the acidic condition in the washing phase of the phage display selection procedure does enhance the chance of discovering neutralizing antibodies.

### The anti-IAV neutralizing antibodies discovered from the synthetic antibody libraries bind to the stem epitopes on the HA trimer

**Figure 8:**
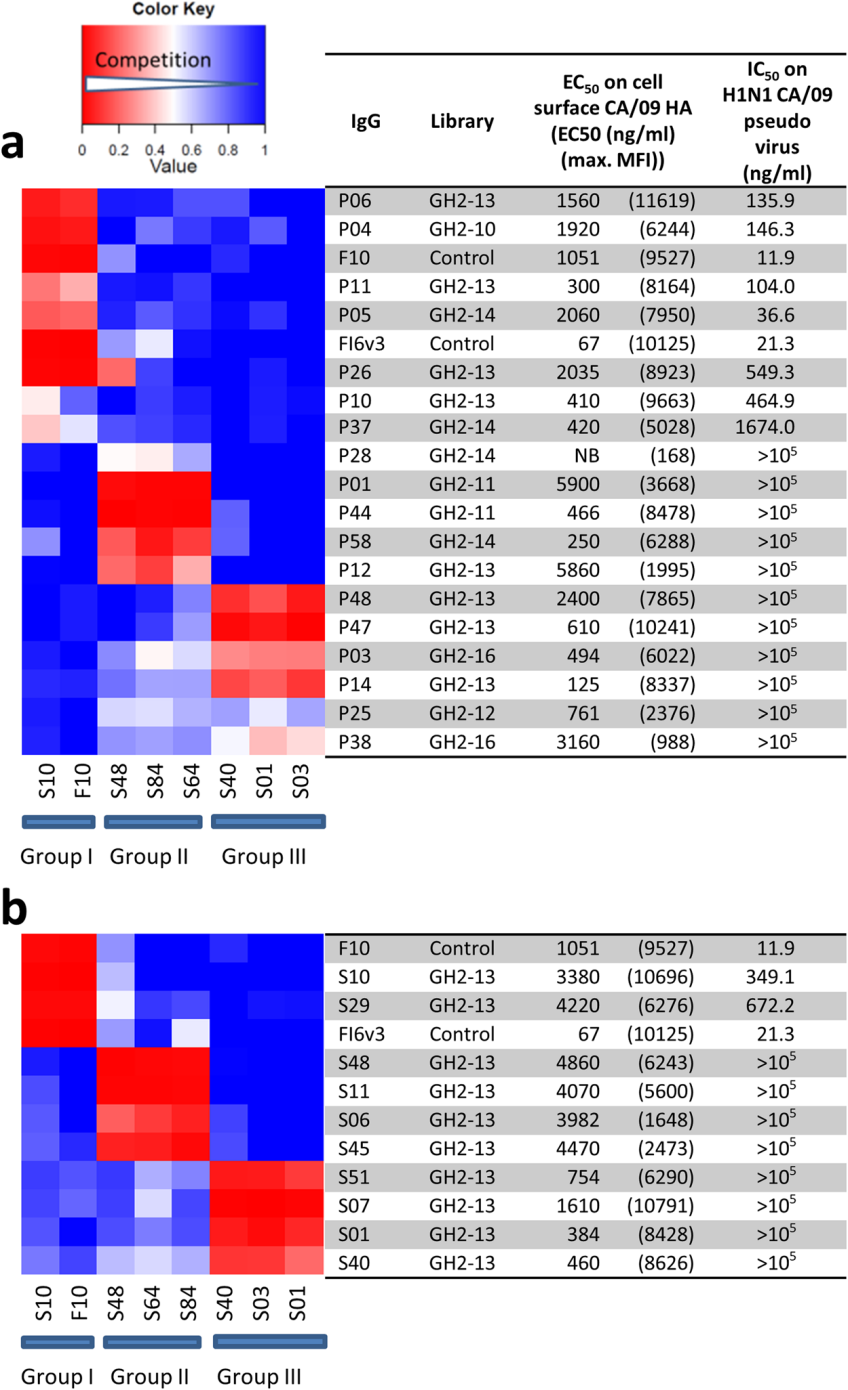
Epitope groups of the anti-HA trimer antibodies determined with competition ELISA. (**a**) Competition between the pairs of antibodies derived from the protocol Step 1a in Fig. 1 are shown as the heat map in this panel. Three major epitope groups (group I, II and III) are distinguishable as shown in the heat map. The names and associated data of these IgGs are shown next to the competition heat map. The experimental details of the competition ELISA are described in Methods. (**b**) The description is the same as in panel A for the competition heat map between the pairs of antibodies derived from the protocol Step 1b in Fig. 1.

The neutralization mechanism of the recombinant IgGs is epitope-dependent (Step 7 in Fig. 1). Competition measurements of the 28 recombinant anti-HA IgGs in Supplementary Table S4 indicate that the epitopes of these IgGs on the H1N1 CA/09 HA trimer can be classified into three major groups (Fig. 8 and Supplementary Table S4). The epitope group classification for the IgGs derived from the Step 1a protocol (Fig. 8a) is strikingly similar to the IgGs derived from the Step 1b protocol (Fig. 8b), indicating that the biopanning protocol and the CDR-H3 sequence length and amino acid composition do not dictate the epitope group distribution of the anti-HA antibodies from the synthetic antibody libraries. Out of the scope of the 28 recombinant anti-HA IgGs in this work, IgG with the group I epitope is a necessary and sufficient condition for the IgG to be an anti-IAV neutralizing antibody; none of the IgGs with epitopes in group II and III are neutralizing antibodies based on the pseudo virus-based micro-neutralization assays (Fig. 8 and Supplementary Table S4). Since both epitopes of the positive control broadly neutralizing antibodies F10^7^ and FI6v3^34^ belong to epitope group I (Fig. 8a,b) and have been known to bind to the stem region of the HA trimer with sub-nanomolar affinity to HA trimers^7,34^, the group I IgGs are anticipated binding to the stem region of the HA trimer with high affinity as well; the stem epitopes have been identified as one of the vulnerable regions on HA trimers targeted by many anti-IAV broadly neutralizing antibodies^26,27,35^.

**Figure 9:**
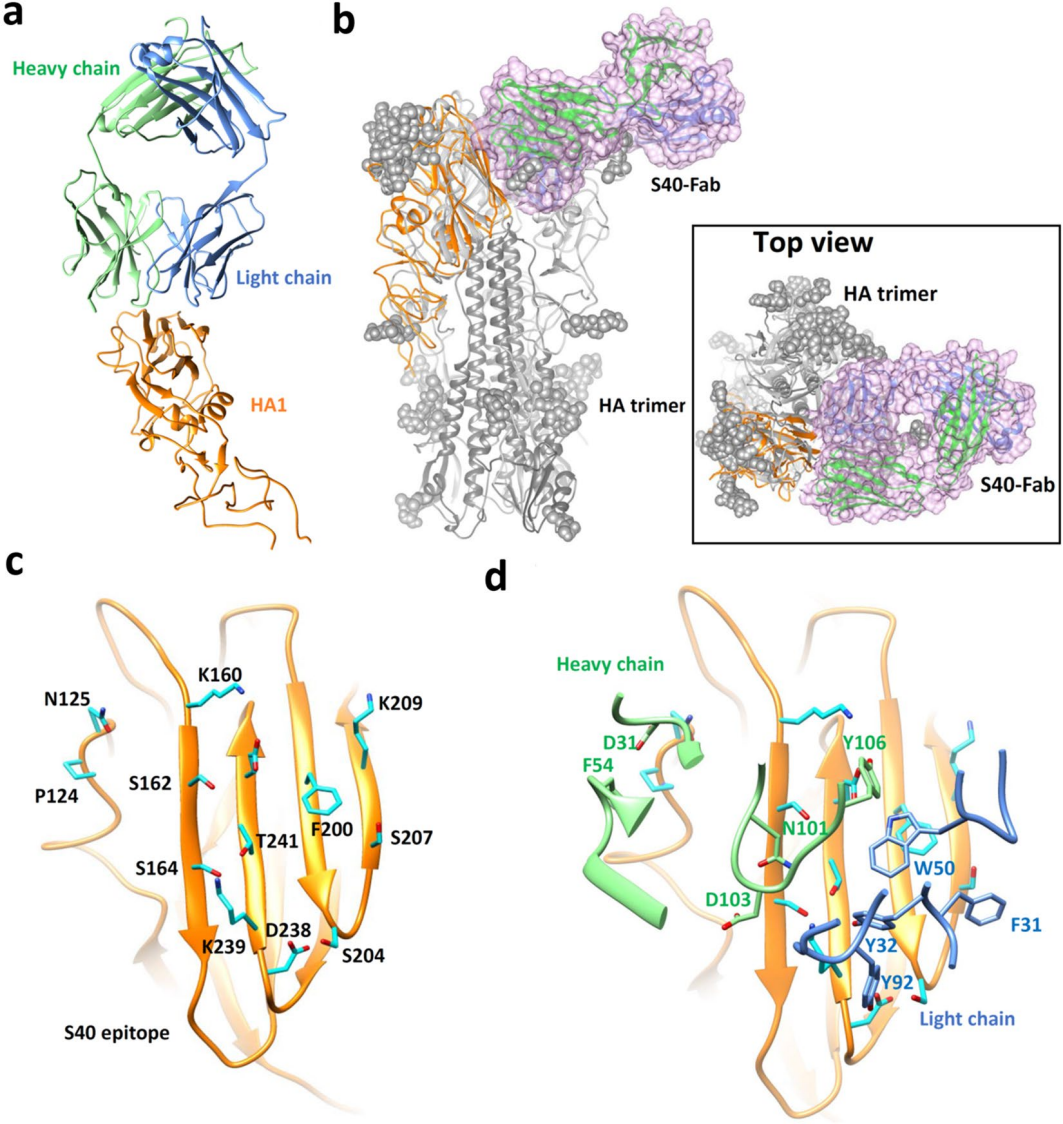
Structure of Fab(S40)-HA(H1N1 CA/09) complex determined with crystallography. (**a**) The Fab(S40) recognizes the HA1 domain as shown by the crystal structure of Fab(S40) in complex with the HA1 domain. The HA1 is colored in orange, the Fab heavy-chain and the light-chain and molecules are in green and blue, respectively. Only the backbone of the complex structure is shown. (**b**) The Fab(S40)-HA1 complex structure is superimposed on the HA trimer (PDB code: 4NRL). The trimeric structure of HA with the glycan (gray sphere) is shown in gray. The HA1 subunit in the Fab(S40)-HA1 complex is shown in orange ribbon and the Fab structure in the Fab(S40)-HA1 complex is outlined by the pink surface. (**c**) Amino acid sidechains in the conformational epitope of S40 on HA1 (backbone shown as the orange ribbon) are shown with stick model in cyan. The residue numbers of these epitope residues are also shown next to the sidechains. (**d**) The heavy-chain and light-chain CDRs in the interface between HA1 and Fab(S40) are shown as green and blue ribbons, respectively. The epitope contacting residue sidechains in the CDRs of Fab(S40) are shown as stick models, which are labeled in green (heavy chain) and blue (light chain) residue numbers.

Antibodies in epitope group III bind to epitopes that are not accessible in pre-fusion HA trimer structure. IgG S40 in group III shows weak neutralization potency against IAV H1N1 CA/09 based on the real virus micro-neutralization assay (Fig. 7 and Supplementary Table S4) and thus was selected for epitope mapping with Fab-HA complex structure crystallography. The Fab(S40)-HA(H1N1 CA/09) complex structure has been solved to 3.28 Å resolution (Fig. 9 and Supplementary Table S5), revealing the binding of the S40 Fab to the inner surface of the HA1 head domain (Fig. 9a) that forms part of the trimer interface and is not accessible as an antibody epitope in the pre-fusion HA trimer structure (Fig. 9b~d). S40 IgG has high affinity to H1N1 CA/09 HA trimer, as indicated by the EC_50_’s based on ELISA and MFI measurements (Figs 4a, 5a and Supplementary Table S4), suggesting that S40 IgG’s epitope on the HA trimer is indeed accessible in both immobilized or cell surface-expressed form of the HA trimer. Moreover, the weak neutralizing potency of the S40 IgG against IAV H1N1 CA/09 (Fig. 7) suggests that the epitope on the HA trimer could be exposed (at least transiently) for S40 antibody binding, leading to the neutralization of the infection by IAV H1N1 CA/09.

## Discussion

Anti-IAV neutralizing antibodies can be readily attained from artificial antibody repertoires. The technologies developed in this work enable a high throughput neutralizing antibody discovery platform that does not necessitate prior memory of antibody-antigen interactions as in natural antibody repertoires with previous exposure to the antigen or rely on affinity maturation essential for *in vivo* immune systems to generate highly specific neutralizing antibodies through rounds of somatic hyper mutation and clonal selection.

Synthetic antibody library designs could lead to diversely novel antibody-protein antigen recognitions that are not found in natural immune systems. The GH synthetic antibody libraries have been established as the core component in a high throughput platform for discovering antibodies, with high specificity and affinity, against protein antigens in general^29,31^. But the utility of the GH-based technological platform had been limited to one synthetic antibody library design (GH2) and for immunotoxin discovery against cancer cells^29,31^. In a previous work on neutralizing antibody discovery against IAV infection^32^, the sequence space of the complementarity determining regions of a broadly neutralizing antibody (F10) targeting the stem epitope on the hemagglutinin of a strain of H1N1 influenza virus had been systematically explored, and the elucidated antibody-hemagglutinin recognition principles were used to design a phage-displayed antibody library, which was then used to discover neutralizing antibodies against another strain of H1N1 virus^32^. But this approach necessitated known antibody-antigen recognition information specific for the parent antibody (F10 in this case) to design and construct phage-displayed antibody libraries, aiming to discover more potent neutralizing antibodies in comparison with the parent antibody. In responding to situations of novel antigens without known neutralizing antibodies, we demonstrate in this work that the general high throughput platform with the expanded GH2-5~24 antibody libraries with diverse CDR-H3 sequence lengths can be used to discover novel neutralizing antibodies against IAV infection in about 4 weeks (Fig. 1), providing passive immunotherapy antibodies as alternatives to neutralizing antibodies derived from natural antibody repertoires. Improvement in the design and construction of the phage-displayed synthetic antibody libraries can further empower the high throughput platform for discovering potent neutralizing antibodies against not only IAV but also other newly emerging viral infections.

At least two of the three epitope groups of the anti-HA antibodies from the GH2 series antibody libraries resemble those of natural anti-HA antibodies. The epitopes in the group I of the GH2 antibodies overlap with those of F10 and FI6v3, which are centered on the helix A of the HA2 domain of the HA trimer. Epitopes in this stem region are frequently targeted by the broadly neutralizing antibodies that are inhibitors of the HA-mediated virus-host cell fusion and are protective against infection with group 1 and group 2 IAV, as well as IBV strains^7,8,34,36,37^, likely because these stem region epitopes are more conserved in amino acid sequence due to the constrain of the pH-dependent fusion mechanism essential to the infection with the influenza virus^26^. The attainment of the neutralizing antibodies from the GH2 series antibody libraries targeting these stem region epitopes (Fig. 8) further emphasizes the feasibility of discovering potent neutralizing antibodies from artificial antibody repertoires.

The group III epitopes of the GH2 antibodies are situated in the anti-parallel beta-sheet surface of the HA1 domain, which forms the interface between the monomers in the intact structure of the HA trimer and is inaccessible for antibody binding due to steric clashes (Fig. 9). A recent study has shown that H1 and H3 cross-reactive antibodies, which are prevalent in the serum of human donors, also recognize this conserved epitope in the HA1 domain. Negative-staining electron microscopy images show that the epitopes of the H1 + H3 cross-reactive antibodies overlap with the group III epitopes of the GH2 antibodies and are also inaccessible in the intact HA trimer. Like the GH2 antibodies in the epitope group III, these HA-head specific H1 + H3 human antibodies do not show neutralization activity *in vitro*. However, these H1 + H3 antibodies are protective in mice against infection with H1N1 and H3N2 virus strains, likely due to effector functions mediated by the Fc domain of the IgGs^38^. These results suggest that the artificial antibodies from the GH2 series antibody libraries are nevertheless relevant to the human antibody responses against the influenza viruses.

The predominant epitopes of the anti-IAV neutralizing antibodies discovered in humans are mostly situated on the surface of the HA1 domain (the head region) accessible in the intact HA trimer, where the antibody binding frequently blocks the host cell receptor sialic acid from binding to the HA trimer, resulting in neutralizing IAV infectivity^26^. These neutralizing antibodies are distinguishable from the fusion inhibition antibodies with hemagglutination inhibition (HAI) assay. None of the GH2 antibodies studied in this work are HAI positive, indicating that the GH2 antibodies targeting the predominant neutralizing epitope on the HA1 domain are rare or cannot be discovered with the current antibody discovery protocol. Antibody library designs targeting the conserved sialic acid binding site on the HA trimer based on the HA structures and sequence information from diverse IAV strains could lead to HAI-positive neutralizing antibodies from the synthetic antibody libraries.

In general, the CDR-H3 residues are known as the main determinants in antibody-protein interactions, and hence we anticipated that the synthetic antibody libraries GH2-5~GH2-24 containing antibody variants with CDR-H3 sequence length ranging from 5 to 24 residues of diverse amino acid types (Supplementary Table S2) should have diverse preferences in epitope locations on the antigen molecules. Surprisingly, CDR-H3 sequence length does not have profound impact on the epitope distributions, as demonstrated in the comparison of the epitope distributions shown in Fig. 8. This result suggests that CDR-L1~H2 sequence designs are at least equally consequential as the designs of CDR-H3 for synthetic antibody libraries with variants aiming to target diverse epitopes on a protein antigen molecule.

In summary, passive immunotherapy with anti-IAV neutralizing recombinant antibodies is one of the viable strategies mitigating threats of epidemic and pandemic IAV infection. Technological platform for high throughput discovery of anti-IAV neutralizing recombinant antibodies is particularly desirable during the outbreak of pandemics when candidate recombinant antibodies need to be rapidly developed before large population infection causing significant mortality, morbidity and economic loss, in particular when affinity matured human antibodies with neutralizing efficacy against the pandemic strain are not available. High throughput screening of functional neutralizing antibodies requires production of thousands monoclonal antibodies in high throughput format with enough concentration for functional assays; the phage-displayed synthetic scFv libraries with scFv expression quality control meet the prerequisite of the high throughput screening for anti-IAV neutralizing recombinant antibodies, as demonstrated in this work. Once the recombinant HA trimer and the pseudo virus of the pandemic IAV strain become available, neutralizing IgG candidates suitable for passive immunotherapy should be attained within one month by following through the high throughput procedure depicted in Fig. 1. With continuing advancement in designing and constructing synthetic scFv libraries containing diverse functional antibody fragments, this technological platform not only provides a promising option in mitigating the threats of IAV pandemics; the technology can also provide options to develop recombinant antibody candidates for passive immunotherapy treating other newly emerging viral infections.

## Methods

### Cell lines

MDCK (Madin-Darby canine kidney, ATCC CCL-34) epithelial cells cultured in MEM medium (Gibco, 11095080) supplemented with NEAA (non-essential amino acids, Gibco, 11140076), 2 mM L-glutamine, and 10% fetal bovine serum at 37 °C in a 5% CO_2_ humidified atmosphere incubator were used to determine microneutralization ability and IC_50_ in this study. 293T cells (ATCC CRL-3216) were cultured in DMEM medium (Gibco, 11965092) supplemented with 10% fetal bovine serum (Gibco, 10437028), penicillin-streptomycin (100×; Gibco, 15140122). Suspension FreeStyle 293-F (293-F, Thermo Fisher Scientific Inc., R79007) cells were cultured in serum free Freestyle 293 expression medium (Gibco, 12338001) at 37 °C with shaking 110 rpm in 8% CO_2_ incubator (Thermo Fisher Scientific Inc.).

### Virus

H1N1 CA/09 influenza A virus is the recombinant virus NYMC X-181 supplied by Taiwan’s CDC. Embryonic eggs (10-day-old) were used to propagate virus stock. H1N1 CA/09 influenza A virus (1000 TCID_50_) was injected into allantoic cavity to amplify virus. After incubation for 60 hours, virus solution was harvested, concentrated by ultracentrifugation (25000 × *g* for 2 hours) and resuspended in PBS^32^.

### TCID50

TCID_50_ (50% tissue culture infectious dose) of H1N1 CA/09 influenza virus titer was determined in MDCK cells with a few modifications^32^. Virus was 10-fold serially diluted in infection buffer (MEM/NEAA medium supplied with 0.3% BSA). PBS-washed MDCK cells (3 × 10^4^ cells per well in a 96-well plate) were infected by the diluted virus solutions for 1 hour and then washed with PBS twice to remove virus solution. Survival MDCK cells were fixed with ice-cold methanol-acetone (1:1 (v/v)) and stained with 0.5% crystal violet 3 days post-infection. Each dilution was performed 8 replicates and the TCID_50_ was calculated according to Reed and Muench method^32^.

### Expression and purification of hemagglutinin (HA) trimer

The cDNAs corresponding to residues 11-329 (HA1) and 1-176 (HA2), based on H3 numbering, of the ectodomain of the H1N1 CA/09, H5N1 VN/04, H3N2 WN/05, and H7N9 AH/13 hemagglutinin (HA) from A/California/07/2009 (H1N1; Accession No. ACP41953.1), A/Vietnam/1203/2004 (H5N1; Accession No. AY651334), A/Wisconsin/67/2005 (H3N2; Accession No. AFH00648.1), and A/Anhui/1-BALF_RG17/2013 (H7N9, Accession No. AHZ59831.1), respectively, were codon-optimized for eukaryotic cell expression and fused to an N-terminal gp67 signal peptide (MLLVNQSHQGFNKEHTSKMVSAIVLYVLLAAAAHSAFA) and to a C-terminal thrombin cutting site, trimerization domain and 6-His-tag (ASLVPRGSPGSGYIPEAPRDGQAYVRKDGEWVLLSTFLGHHHHHH) by PCR as described previously^8^. These HA expression cassettes were inserted into pFastBac-1 (Invitrogen, 10360014), a baculoviral transfer vector. HA protein was produced by infecting suspension cultures of SF9 cells (3 × 10^6^ cells/mL) (ATCC CRL-1711) with recombinant baculovirus at an MOI of 5 and incubated at 27 °C with shaking at 110 rpm for 72 hours. The cultures were clarified by two rounds of centrifugation (1000 × g and 12000 × g for 30 minutes, 4 °C, respectively). The supernatants containing HA was filtered by 0.8 μm pore size filter before purification. The HA was purified by Ni^2+^-covalent bound HisTrap excel prepacked column (GE Healthcare Life Sciences, 17-3712-06) by gradient from 10 mM to 500 mM imidazole in TS solution (Tris-HCl 20 mM, NaCl 50 mM, pH 8.0). The fractions containing HA were introduced to Q column chromatography (GE Healthcare Life Sciences, 17-1154-01) and eluted by gradient from 50 mM to 1000 mM NaCl in Tris-HCl 20 mM, pH 8.0. The fractions containing HA were concentrated and introduced to Superdex 200 10/300 GL column (GE Healthcare Life Sciences, 17517501) for size exclusion chromatography with TS solution. The fractions containing HA were collected and stored in 4 °C or −80 °C. For crystallization, the HA was digested with thrombin (0.2 unit per mg HA at 4 °C overnight) to remove the trimerization domain and 6-His-tag. The digested materials were purified with Superdex200 10/300 GL column with TS solution directly. The purified monomeric HA was mixed with 1.5 molar ratio of S40 Fab at 4 °C overnight with gently shaking. The HA-Fab complex was purified with Superdex200 10/300 GL column with TS solution to remove excess Fab.

### Phage-displayed scFv library construction

The construction and characterization of the phage-displayed synthetic scFv libraries followed the same procedure, without modification, as described in the previous work^29^. The library construction method is described in detail in Supplementary Methods. The CDR-L1~H3 sequences for the GH2-5~24 scFv libraries are shown in Supplementary Tables S1~S2. The Illumina MiSeq NGS sample preparation and data analysis followed the same procedures as described in previous works^39,40^. The experimental procedures for selection of specific scFv binders, microneutralization assay, IC_50_ determination, IgG reformatting from scFv, IgG expression and EC_50_ determination for antibody-antigen interaction have been described in previous works^29,31,32,39,40^. Minor modifications are described below:

### Selection and screening of anti-HA monoclonal scFvs from phage-displayed scFv libraries

#### Phage display selection-amplification cycles

Recombinant H1N1 CA/09 HA (10 μg per well) was coated on Nunc 96-well Maxisorb immunoplates (Nunc 442404), and then blocked with 5% skim milk in PBST for 1 hour. After blocking, 100 μL of resuspended polyethylene glycol/NaCl-precipitated phage library (10^13^ cfu/mL in blocking buffer) was added to each well for 1 hour under gently shaking. The plate was washed 12 times with 200 μL PBST [1 × PBS + 0.05% (v/v) Tween 20, pH 7.4] and 2 times with 200 μL PBS (Step 1b in Fig. 1). The bound phages were eluted with 100 μL of 0.1 M HCl/glycine (pH 2.2) per well, immediately neutralized with 8 μL of 2 M Tris-base buffer (pH 9.1). The eluted phages were mixed with 1 mL of *E. coli* ER2738 (A_600 nm_ = 0.6) for 30 minutes at 37 °C; uninfected bacteria were eliminated by adding ampicillin. After ampicillin treatment for 30 minutes, the bacterial culture was infected with 100 μL M13KO7 helper phage (~10^11^ CFU total, New England Biolabs, N0315S) at 37 °C for 1 hour, and then added to 50 mL of 2 × YT medium containing kanamycin 50 μg/mL and ampicillin 100 μg/mL overnight at 37 °C with vigorously shaking. The rescued phage library was precipitated with 20% polyethylene glycol/NaCl, and resuspended in PBS. The concentrated phage solution was used for the next round of panning. In the biopanning procedure with acidic washing phase (Step 1a of Fig. 1), the plate was washed 12 times with 200 μL mildly acidic buffer PBST [1 × PBS pH5.0 + 0.05% (v/v) Tween 20] and 2 times with 200 μL PBS (pH7.4) in the second and third round of biopanning. After the washing phase, the phages were eluted and rescued as described above.

#### Ratio of output/input phage library titer

In each biopanning procedure as described above, the output (eluted) and input phage were tittered with fresh-prepared *E. coli* ER2738, and the ratio of output/input titer was calculated.

#### Polyclonal soluble scFvs in *E. coli* culture media evaluated for antigen binding with ELISA

50 μL rescued phage from each cycle of biopanning above was mixed with 750 μL of *E. coli* ER2738 (A_600 nm_ = 0.6) in 96-well deep well culture plate and incubated at 37 °C with vigorously shaking. One hour later, 100 μL ampicillin was added to final concentration 100 μg/mL ampicillin. 100 μL of 10 mM IPTG was added to each well (final concentration 1 mM) after A_600 nm_ > 1.0, and the plate was incubated at 37 °C with vigorously shaking overnight. The plate was centrifuged at 3000 × g for 10 minutes and the supernatants were used for ELISA binding assay below.

#### ELISA assay for soluble scFv-antigen binding

After 2–3 rounds of selection-amplification cycle, single colonies were picked and soluble monoclonal scFvs secreted in the *E. coli* cultures were prepared^29^. Nunc 96-well Maxisorb immunoplate coated with CA/09 H1 HA 0.5 μg per well was blocked with 5% skim milk in PBST [0.05% (v/v) Tween 20] for 1 hour. 100 μL cultured medium with secreted scFv was added to the plate for binding. After 1 hour of binding and washing six times with PBST, 100 μL anti E-tag-HRP (1:4000 dilution, ICL Inc., RET-45P) was added to each well. After 1 hour incubation, the plate was washed six times with PBST buffer and twice with PBS, developed for 3 minutes with 3,3′,5,5′-tetramethyl-benzidine peroxidase substrate (TMB substrate, Kirkegaard & Perry Laboratories, 52-00-04), quenched with 1.0 M HCl and read spectrophotometrically at 450 nm.

#### ELISA assay for soluble scFv folding with Protein L/Protein A

In additional to test the antigen binding of secreted scFv, well-folded scFvs were identified with Protein L and Protein A binding. Nunc 96-well Maxisorb immunoplate coated with Protein L (Thermo Fisher Scientific Inc., 21189) 0.1 µg per well was blocked and added with scFv cultured medium as described above. The signals were developed with Pierce Protein A conjugated with horseradish peroxidase (1:5000 dilution, Thermo Fisher Scientific Inc., 101023).

### Microneutralization assay

MDCK cells (3 × 10^4^ cells/well) were seeded in 96-well plates for 16 hours and washed twice with PBS prior to be infected by virus-scFv mixture. Filtrated scFv solution was freshly mixed with 100 TCID_50_ viral solution (1:1 (v/v)) and incubated for 1 hour at 37 °C. Virus-scFv mixtures were then added to infect PBS-washed MDCK cells for another 1 hour at 37 °C. Virus-scFv mixtures were removed and cells were washed twice with PBS after absorption. Infected MDCK cells were cultured in infection buffer 24 hours post-infection and then fixed with methanol-acetone (1:1 (v/v)). Staining procedure followed the procedure in previous publication^32^. Each filtrated scFv was assayed once in a 96-well plate with duplicate and every test was performed with two independent experiments. The relative viral activity was calculated with ELISA readings according to the previous publication with some modification^32^. The ELISA readings of virus-only control and that of negative control (no virus and no scFv) were set as 100% and 0% respectively. The neutralization percentage was calculated by the reduction of virus infection due to scFv addition.

### **IC**_**50**_**determination**

MDCK cells were used to determine the half of maximal inhibitory concentration (IC_50_) of purified IgG candidates^32^. In brief, a serial 2-fold diluted IgG was mixed with 100 TCID_50_ viral solution and incubated for 1 hour at 37 °C. PBS-washed cells were infected by virus-IgG solution for another 1 hour at 37 °C. Staining procedure followed the procedure in previous publication^32^. Mouse anti-influenza A viral nucleoprotein IgG antibody (1:2000 dilution, Millipore, MAB8251) and goat anti-mouse antibody conjugated with HRP (1:1000 dilution, Millipore 12-349) were used to detect propagated viruses. TMB substrate (100 µL per well) was added and the absorbance at 450 nm was measured after reactions were stopped by adding 1 N HCl (100 µL per well). Each concentration of diluted IgG was assayed with triplicate. The IC_50_ (ng/mL) was calculated as previously published^32^.

### IgG reformatting and expression from scFv

As depicted in step 5 of Fig. 1, variable domain of light chain (VL) and heavy chain (VH) gene of a scFv in a phagemid were cloned into IgG expression plasmid, pIgG (U.S. patent No. 5736137), to express secreted human IgG. pIgG vector was linearized with *Nhe*I and *Kpn*I restriction enzymes. VL and VH genes were PCR amplified separately, assembled into a 1.8Kb fragment and infused into linearized pIgG vector with Gibson Assembly Kit (New England BioLabs, E2611L). The pIgG expression vector was transfected into 293-F cell; the secreted IgG in the culture medium was purified with Protein A agarose (Thermo Fisher Scientific Inc., 20334). More detailed methods in IgG construction, plasmid transfection, and IgG purification have been described in our previously publication^29^. The positive control IgGs: F10^7^, FI6v3^34^, C05^41^, and CR8020^42^ were expressed and purified with the same method described in this section.

### EC_50_ for antibody-antigen interaction

The IgG EC_50_ was determined by the titrations of IgG antibody on immobilized recombinant HA protein with ELISA. Four HA antigens, including H1N1 CA/09, H3N2 WN/05, H5N1 VN/04 or H7N9 AH/13 HA (0.5 µg per well) were coated in PBS buffer (pH7.4) on Nunc 96-well Maxisorb immunoplates. Detailed methods of EC_50_ determination were described in our previously publication^29^. EC_50_ and sigmoidal curve fitting correlation coefficients were calculated using the ED50 Plus v1.0 Excel worksheet developed by Dr. Mario H. Vargas at Instituto Nacional de Enfermedades Respiratorias (http://www.sciencegateway.org/protocols/cellbio/drug/data/ed50v10.xls). IgGs with the ELISA readings of 10 µg/mL IgG in the range of background (OD_450nm_ = 0.04–0.05) were assigned as non-binding (NB).

### Influenza pseudo virus-based microneutralization assay

The H1N1 CA/09, H3N2 WN/05, or H7N9 AH/13 pseudo virus produced by co-transfection lentiviral core plasmid encoding luciferase (pHR’CMV-Luc), pCMV-R8.2 encoding HIV Gag-Pol, plasmids encoding TMPRSS2, respectively H1 A/California/04/2009 (Accession No. ACP41953.1), H3 A/Wisconsin/67/2005 (Accession No. AFH00648.1), H7 A/Anhui/1-BALF_RG17/2013 (Accession No. AHZ59831.1), and corresponding neuraminidase (pHR’CMV-Luc, pCMV-R8.2, plasmids expressing TMPRSS2, H1 A/California/04/2009, and N1 A/California/04/2009 were kind gift from Drs. Barney Graham and Michelle Crank (NIH/NIAID)). H5N1 pseudo virus was produced by co-transfection plasmid pLN4_3.Luc.R-E-, plasmids encoding H5 A/Vietnam/1203/2004 (Accession No. AY651334), and N1^43^. After transfection overnight, cells were replenished with fresh medium and incubated for another 48 hours. Then the supernatant were collected, filtered through 0.45 μm syringe filter, and stored at −80 °C until use. Microneutralization of pseudo virus was performed by incubated serial diluted antibodies with 100 TCID_50_ pseudo virus for 45 minutes and then infect 1 × 10^4^ 293T cells in a well of 96 well plate. The medium was replenished with fresh medium 16–18 hours after infection. Sixty hours after infection, cells were lysed by 20 μL 1 × lysis buffer (Promega, E2661) per well and mixed with shaking for 15 minutes. Luciferase reagent (Promega, E2650) was added to each well of white 96 well microplate (Griener Bio-one, 655075). Cell lysate was transferred to corresponding well of white 96 well microplate prior to analysis of luciferase activity by Victor3 (Perkin Elmer). The luciferase signal of virus-only control and that of negative control (no virus and no scFv) were set as 100% and 0% respectively. The neutralization percentage was calculated by the reduction of virus infection due to scFvs neutralization. Each concentration of diluted IgG was assayed with at least two repeats. The IC_50_ (ng/mL) was calculated as previously published^32^.

### Flow cytometry assay of antibody binding to HA-expressing stable transfectants

H1 A/California/07/2009 (Accession No. ACP41953.1) and H5 A/Vietnam/1203/2004 (Accession No. AY651334) were cloned into pLAS2.PeGFP.Puro. which express green fluorescence protein (GFP) and puromycin resistant gene. HA-expressing stable transfectants were carried out by transfecting 293 T cells separately with the full-length Influenza A pLAS2.H1 A/California/07/2009.PeGFP.Puro and pLAS2.H5 A/Vietnam/1203/2004.PeGFP.Puro. with PEI (Polysciences Inc., 24765). Forty-eight hours post transfection, transfectants were selected with 2 μg/mL puromycin (Sigma-Aldrich, P8833) to obtained mixed stable transfectants. To analyze antibody binding to native HA protein, we used 293 T H1 and H5 stable transfectants stained with serial diluted IgGs at 4 °C for 30 minutes. Following the incubation, the cells were washed once with FACS buffer (1 × PBS + 0.05% FBS) and stained with the Alexa-Fluor 633-conjugated goat anti-human IgG (Thermo Fisher Scientific Inc., A-21091) secondary antibody. The cells were then incubated at 4 °C for another 30 minutes. After washing with FACS buffer twice, APC fluorescence signal of GFP^+^ cells was analyzed by FACSCANTO II flow cytometer (Becton Dickinson Immunocytometry Systems) equipped with FACSDiva™ software (Becton Dickinson). Cells incubated with isotype negative control antibody were measured as negative control. Mean fluorescence intensity (MFI) of 10,000 GFP^+^ cells was determined by FACSDiva™ software. Maximal MFI is the highest MFI in serial concentration of IgGs staining. EC_50_ were determined with the Prism software (GraphPad Software Inc.). Mean fluorescence intensity (MFI) below 2.5 times of Do1 negative control IgG (Supplementary Tables S3 and S4) in independent experiment was defined as non-binding (NB).

### Competition of antibody-HA interaction

To investigate the binding epitopes of selected anti-HA scFvs, we used a modified phage ELISA to detect the competition of the scFvs binding to recombinant HA with a panel of purified anti-HA IgGs. Test phages were fresh prepared. H1N1 CA/09 HA antigen (0.5 µg per well) were coated in PBS buffer (pH7.4) on Nunc 96-well Maxisorb immunoplates overnight at 4 °C, and blocked with 5% skim milk in PBST[0.05% (v/v) Tween 20] for 1 hour. After blocking, 1~3 µg purified anti-HA IgG was added to each well for 30 minutes under gently shaking and then added 50 µL test phage for another hour incubation. The plate was washed 6 times with 300 µL PBST [0.05% (v/v) Tween 20] and incubated 1 hour with horse-radish peroxidase/anti-M13 antibody conjugate (1:3000 dilution, GE Healthcare, 27-9420-01). The plates were washed six times with PBST buffer and twice with PBS, developed for 5 minutes with TMB substrate, quenched with 1.0 M HCl and read spectrophotometrically at 450 nm. Competition values were calculated by comparing each control sample without adding anti-HA IgGs. For competition analysis, the gplots package of R software (http://www.r-project.org/) was used for generating the heat map with a dendrogram for the competition data where the competition values were normalized from 0 to 1.0.

### S40 Fab expression and purification

S40 was expressed in mammalian cells (293-F cells) as a 6-His-tagged Fab with PEI transfection. The Fab expression vector was derived from the IgG expression plasmid, pIgG (U.S. patent No. 5736137), which did not contained CH2 and CH3 domains of heavy chain but with an additional 6-His-tag at C-terminus of CH1 domain of heavy chain, which forms disulfide bond with the constant domain of the light chain. Variable domain of light chain (VL) was subcloned into *Kpn*I site while variable domain of heavy chain (VH) domain was subcloned into *Nhe*I site of the pIgG plasmid. S40 Fab was expressed by 293-F cells. 293-F cells were cultured to a final 1–1.5 × 10^6^ cells/mL in Freestyle 293 expression medium (Gibco, 12338001), and incubated for 2–4 hours at 37 °C (8% CO_2_, 110 rpm). S40 Fab construction was transfected into 293-F cells with PEI (Polysciences Inc., 24765) according to supplier’s protocol. Expressed S40 Fab was harvested from the supernatant 7–9 days or until cell viability drops below 60% after transfection. The S40 Fab was purified by Ni^2+^-covalent bound HisTrap excel prepacked column by gradient from 10 mM to 500 mM imidazole in TS solution (Tris-HCl 20 mM, NaCl 50 mM, pH 8.0). The fractions containing S40 Fab were concentrated and introduced to Superdex200 10/300 GL column for size exclusion chromatography with TS solution. The concentration of purified S40 Fab was measured by optical absorbance at 280 nm, and the purity and integrity was analyzed by reducing and non-reducing SDS-PAGE. The fractions containing S40 Fab were collected and stored in 4 °C or −80 °C.

### Crystallization and data collection

Crystals of HA1/S40-Fab complex were grown by mixing 1 µL protein solution with 1 µL reservoir solution using the sitting-drop vapor-diffusion method at 293°K. The crystals were obtained in a reservoir solution consisting of 8% (w/v) PEG 6000, 1 N NaOH, 15% MPD, 0.1 M Na HEPES, pH 7.5. All crystals were flash-cooled and the diffraction patterns were recorded at cryogenic temperatures. The diffraction data of HA/S40-Fab complex crystals were collected at a wavelength of 0.90 Å on beamline BL44XU of the SPring-8 synchrotron in Japan using an MX-225 CCD detector. Diffraction data were processed and scaled to 3.35 Å resolution using HKL-2000^44^.

### Structure determination and refinement

The HA1/S40-Fab complex crystal structure was determined by molecular replacement using MOLREP from the CCP4 suite^45^ with the IgG1 domain fragments (PDB entry 3W9D and 3HC4) and the HA1 fragment (PDB entry 3GBN) as the search models. The HA/S40-Fab complex crystals belong to space group *C*2, with two HA1/S40-Fab complexes in an asymmetric unit. Throughout the refinement, a randomly selected 5% of the data were set aside for cross-validation by the *R*_free_ value. Manual modifications of the models were performed using the program Coot^46^. The complex structure was refined using REFMAC5^47^, from which *R*_work_ and *R*_free_ values of 23.0 and 27.5%, respectively, were obtained. Data-collection and final model statistics are shown in Supplementary Table S5. The molecular figures were produced using UCSF Chimera^48^. The atomic coordinates and structure factors of the crystal structures of HA1/S40-Fab complex have been deposited in the Protein Data Bank with accession code 5XHV.

### Ethics statement

All methods were carried out in accordance with relevant guidelines and regulations. All experimental protocols were approved by Academia Sinica Biosafety Review & Biomaterials and Lab Biosafety Information System (BSF0414-00002956). Specific pathogen free eggs were purchased from Animal Health Research Institute in Taiwan. Embryonic eggs (10-day-old) were inoculated to propagate virus stock. Approval from an animal ethics committee does not required according to IACUC Policy^49^.

### Data Availability

The Authors confirm that all relevant data are included in the paper and/or its supplementary information files.

## Electronic supplementary material

Supplementary information
