# Supplementary material for: High throughput discovery of influenza virus neutralizing antibodies from phage-displayed synthetic antibody libraries
Source: Sci Rep. 2017 Oct 31;7:14455. doi: 10.1038/s41598-017-14823-w (PMC5663709; doi:10.1038/s41598-017-14823-w)
Supplement: Supplementary file 1 — Supplementary information [file 41598_2017_14823_MOESM1_ESM.pdf]

Supplementary information

High throughput discovery of influenza virus neutralizing antibodies from phage-displayed synthetic antibody libraries

Ing-Chien Chen[1,+], Yi-Kai Chiu[1,+], Chung-Ming Yu[1], Cheng-Chung Lee[2], Chao-Ping Tung[1], Yueh-Liang Tsou[1], Yi-Jen Huang[1], Chia-Lung Lin[1], Hong-Sen Chen[1], Andrew H.-J. Wang[2], An-Suei Yang[1,*]

[1] Genomics Research Center, Academia Sinica, Taipei, Taiwan 115.

[2] Institute of Biological Chemistry, Academia Sinica, Taipei, Taiwan 115.

*Correspondence and requests for materials should be addressed to A.S.Y. (email: yangas@gate.sinica.edu.tw). Genomics Research Center, Academia Sinica, 128 Academia Rd., Sec.2, Nankang Dist., Taipei, Taiwan 115. Phone: +886-2-2787-1232.

[+] These authors contribute equally to this work.

Inventory of Supplemental Information

Supplementary Methods

Generic Human (GH) synthetic antibody library construction

Supplementary Figure

Supplementary Figure S1. Sequence preferences determined with NGS for the VH designed positions at different panning stage against HER2 for the selected scFv libraries.

Supplementary Figure S2. Results of pseudo virus-based microneutralization assay for the IgGs that did not shown neutralization activity.

Supplementary Figure S3. SDS-PAGE analysis of purified IgGs.

Supplementary Tables

Supplementary Table S1. Primers for diversifying CDR-L1, L2, L3, H1, H2 in GH2-5~24 antibody libraries.

Supplementary Table S2. Summary of CDR-H3 designs for GH2-5~24 phage-displayed synthetic scFv libraries.

Supplementary Table S3. CDR sequences of anti-influenza IgGs derived from synthetic antibody libraries

Supplementary Table S4. Protein expression, epitope grouping and assessment of the neutralizing and binding potencies of anti-influenza IgGs derived from synthetic antibody libraries

Supplementary Table S5. Crystallography parameters for the S40-HA complex structure

References

Supplementary Methods

Generic Human (GH) synthetic antibody library construction

scFv template preparation: The framework sequence of GH2-5~24 scFv libraries is based on the human IGKV1-NL1*01/IGHV3-23*04 germline sequence and cloned into pCANTAB5E (GE Healthcare) phagemid via *Sfi*I and *Not*I restriction sites. TAA stop codons were introduced in CDRs to ensure that only the phagemids carrying the mutagenic oligonucleotides would produce pIII fusion scFv on phage surface.

Primer design and heavy chain/light chain variable domain library construction: A phage displayed library for each of the GH2-5~24 libraries' light and heavy chain was constructed based on the oligonucleotide-directed mutagenesis procedure [1]. Positions were mutagenized using synthesized oligonucleotides with the following degenerate codons to produce equal molar ratio of designed amino acids: Trp/Gly ([T/G]GG), Phe/Ser/Tyr (T[T/C/A][C/T], Gly/Asp/Ser/Gln ([G/A][G/A][C/T]), Gly/Ala/Ser/Thr/Arg/Pro ([G/A/C][G/C][T/C]), Ala/Thr/Pro/Ser ([A/G/T/C]C[A/G/T/C]), Phe/Tyr/Asp/Val/Asn/Ile/His/Leu ([A/G/T/C][A/T][T/C]), and Leu/Ile/Val/Phe/Met ([A/G/T/C]T[A/G/T/C]) (Supplementary Table S1 and S2). For the light chain repertoires, CDR-L1, -L2 and -L3 were diversified with the mutagenic oligonucleotides shown in Supplementary Table S1 on the basis of the template V3a-LC TAA [2]. For the heavy chain repertoires, CDR-H1, -H2 and -H3 were diversified with the mutagenic oligonucleotides shown in Supplementary Table S1 (CDR-H1 and -H2) and Supplementary Table S2 (CDR-H3) on the basis of the template V3c-HC TAA [2]. In brief, mutagenic oligonucleotides for each CDR were mixed and phosphorylated by T4 polynucleotide kinase (New England BioLabs) in 70 mM Tris–HCl (pH 7.6), 10 mM $MgCl_2$, 1 mM ATP and 5 mM dithiothreitol (DTT) at 37℃ for 1 h. The phosphorylated oligonucleotides were then annealed to uracilated single-

stranded DNA template, at a molar ratio of 3:1 (oligonucleotide:ssDNA), by heating the mixture at 90 ℃ for 2 min, followed by a temperature decrease of 1℃/min to 20 ℃ in a thermal cycler. Subsequently, the template-primer annealing mixture was incubated in 0.32 mM ATP, 0.8 mM dNTPs, 5 mM DTT, 600 units of T4 DNA ligase, and 75 units of T7 DNA polymerase (New England BioLabs) to prime *in vitro* DNA synthesis. After overnight incubation at 20 ℃, the synthesized dsDNA was desalted and concentrated by a centrifugal filter (Amicon® Ultra 0.5 mL 30K device), then electroporated into *Escherichia coli* ER2738 at 3000 V with an electroporator. Typically, 1 µg of dU-ssDNA produced about 10^7-10^8 recombinant phage variants, and 75–90% of the phage variants carried mutagenic oligonucleotides at the three CDR regions simultaneously.

Protein A/L selection of functional scFv variants: The rescued phage libraries of light- and heavy-chain were precipitated with 20% PEG/NaCl and resuspended in phosphate-buffered saline (PBS) for the following protein A/L selection process. First, NUNC 96-well Maxisorb immunoplates were coated overnight at 4 ℃ with Protein A (for selection of heavy chain-diversified libraries) or Protein L (for selection of light chain-diversified libraries) (1 µg/100 µL PBS per well) and blocked with 5% skim milk in PBST for 1h. After blocking, 100 µL of resuspended phage library (10^{13} cfu/mL) was added to each well for 1 h under gentle shaking. The plate was washed 12 times with 200 µL PBST [0.05% (v/v) Tween 20] and 2 times with 200 µL PBS. The bound phages were eluted with 100 µL of 0.1 M HCl/glycine (pH 2.2) per well, followed by neutralization with 8 µL of 2 M Tris-base buffer (pH 9.1). The eluted phages were mixed with 1 mL of *E. coli* strand ER2738 ($A_{600\ nm}$ = 0.6) for 15 min at 37 °C. Infected *E. coli* was titered, and amplified with 50 mL of 2 X YT containing 100 µg/mL

ampicillin at 37 °C overnight. After centrifugation, the bacterial pellet was resuspended and its phagemid DNA was extracted.

Combination of functional scFv variants into the generic human (GH) antibody libraries: Each of the GH2-5~24 libraries was assembled in scFv format as previously described with some modification [3]. In the first PCR, two variable domains VL and VH were amplified separately from light- and heavy-chain library after selection for binding to Protein A/L by using the primers V_L*for* (5’-GGGCCCAGCCGGCCATGGCCGATATTCAAATGACCCAGAGCCCGAGC-3’) with V_L*rev* (5’-GGAAGATCTAGAGGAACCACCGCGTTTGATTTCCACTTTGGTGCCTTGACC -3’) and V_H*for* (5’-GGTGGTTCCTCTAGATCTTCCTCCTCTGGTGGCGGTGGCTCGGGCGGTGGT GGGGAAGTGCAGCTGGTGGAATCGGG -3’) with V_H*rev* (5’-CCTGCCTGCGGCCGCTGACGCCGAGC -3’), respectively (linker sequence is underlined). PCR reactions were performed in a volume of 50 µL using KOD Hot Start polymerase (Novagen), 100 ng DNA template and 0.3 µM of each primer for 25 cycles (30 sec 95°C, 30 sec 65°C, 1 min 72°C) followed a 10 min final synthesis step. The PCR products were digested with *EcoR*I and then purified by agarose gel electrophoresis. In the second PCR, two variable domains were assembled using the overlapping primers (*Sfi*I and *Not*I restriction sites are underlined): *Overlapfor* (5’-GAGGAGGAGGAGGAGGAGGCGGGGCCCAGCCGGCCATGGCCGATATTC -3’) with *Overlaprev* (5’-GAGGAGGAGGAGGAGGAGCCTGCCTGCGGCCGCTGACGCC -3’). 100 ng of the purified VL and VH PCR products of the first PCR were used in a a volume of 50 µL using MyTaq Hot Start polymerase (Bioline) and 0.3 µM of each primer for 30

cycles (30 sec 95°C, 30 sec 65°C, 1 min 30 sec 72°C) followed by a 10 min final synthesis step. The assembled VL-VH fragments were doubly digested with *Sfi*I and *Not*I (New England BioLabs) and cloned into pCANTAB5E phagemid vector. The resulting ligation product was electroporated into *Escherichia coli* ER2738 at 3000 V with an electroporator.

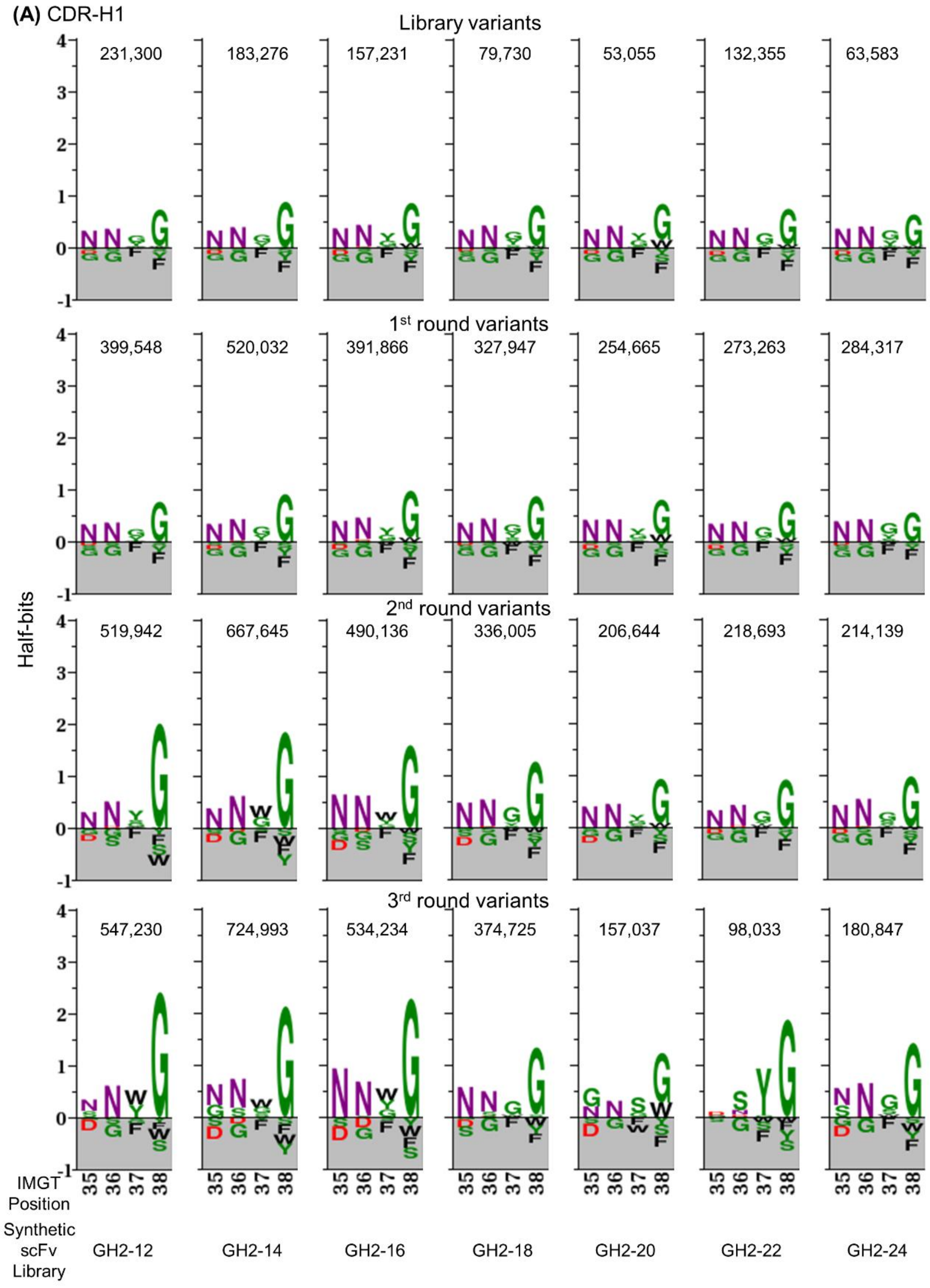
(A) CDR-H1
Library variants
231,300
183,276
157,231
79,730
53,055
132,355
63,583
1st round variants
399,548
520,032
391,866
327,947
254,665
273,263
284,317
2nd round variants
519,942
667,645
490,136
336,005
206,644
218,693
214,139
3rd round variants
547,230
724,993
534,234
374,725
157,037
98,033
180,847
Half-bits
IMGT Position
35 36 37 38
Synthetic scFv Library
GH2-12
GH2-14
GH2-16
GH2-18
GH2-20
GH2-22
GH2-24

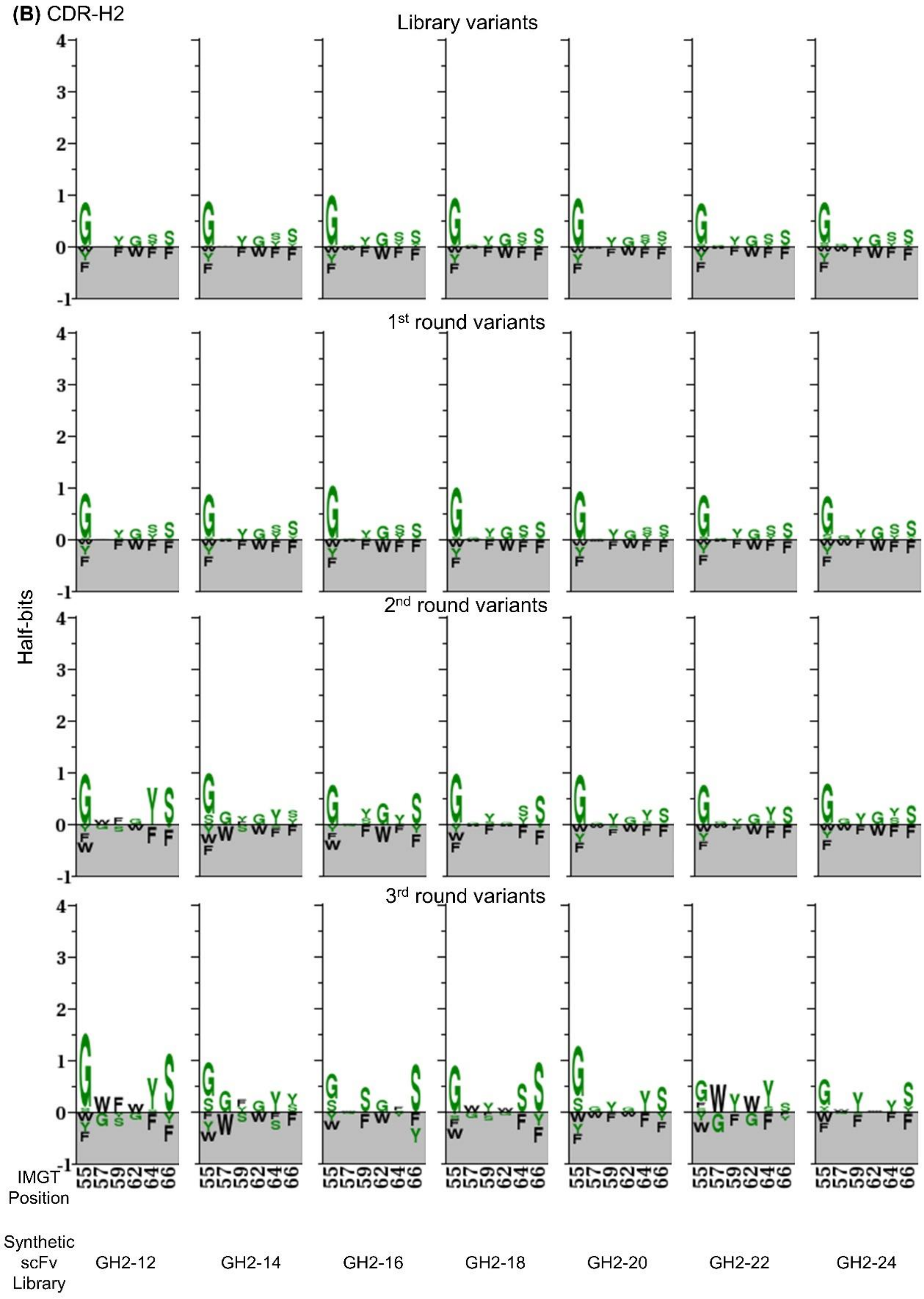
(B) CDR-H2
Library variants
1st round variants
2nd round variants
3rd round variants
Half-bits
IMGT Position
55 57 59 62 64 66
Synthetic scFv Library
GH2-12
GH2-14
GH2-16
GH2-18
GH2-20
GH2-22
GH2-24

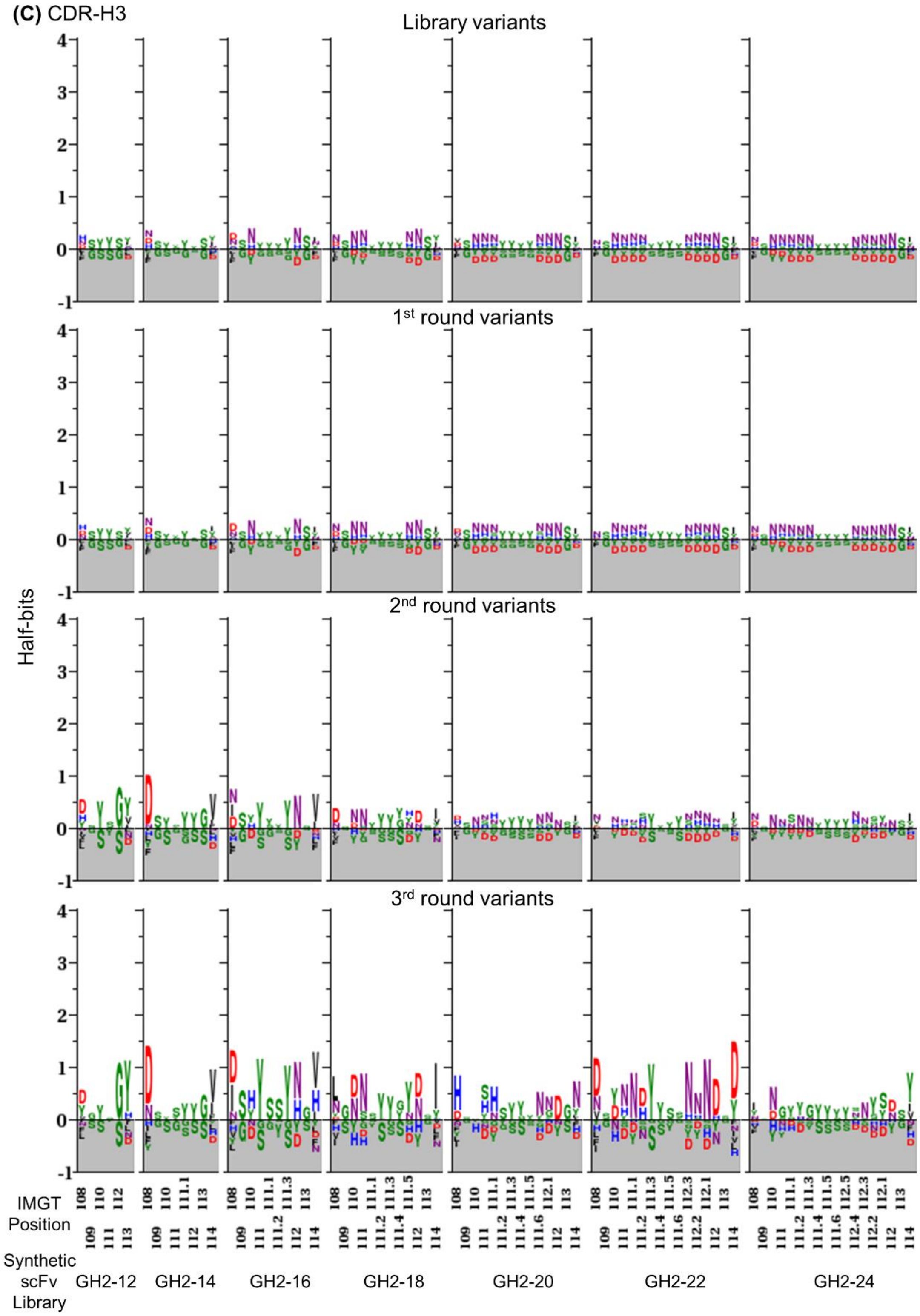
(C) CDR-H3
Library variants
1st round variants
2nd round variants
3rd round variants
Half-bits
IMGT Position
Synthetic scFv Library
GH2-12
GH2-14
GH2-16
GH2-18
GH2-20
GH2-22
GH2-24

Supplementary Figure S1. Sequence preferences determined with NGS for the VH designed positions at different panning stage against HER2 for the selected scFv libraries. (A)~(C) Sequence preference of CDR-H1, CDR-H2, and CDR-H3 respectively. The scFv variants from initial designed library and 3 rounds panning against HER2/ECD are collected for high-throughput sequencing using Illumina MiSeq platform. The number of sequences derived for each group is shown on the top of sequence logo in (A). The calculation of sequence logo is described in previous publication [4]. The background frequency at each position is based on the design (See Supplementary Table S1[2] for CDR-H1 and CDR-H2 design details and Supplementary Table S2 for CDR-H3 design details). CDR position numbering follows IMGT numbering.

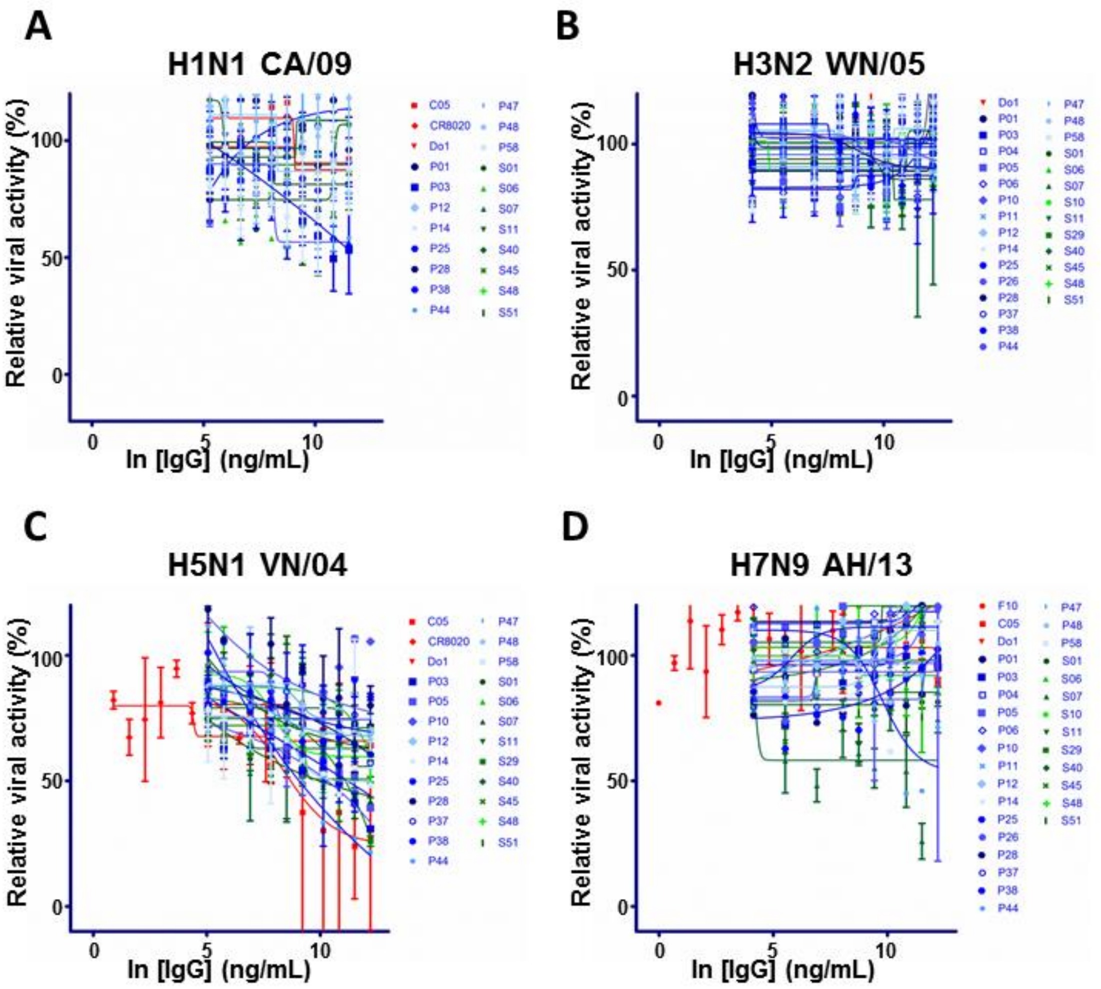

Supplementary Figure S2. Results of pseudo virus-based microneutralization assay for the IgGs that did not shown neutralization activity. (A)~(D) The IgGs were reformatted from the selected anti-HA trimer scFvs as shown in Fig. 3. The y-axis shows the relative viral activity plotted against the IgG concentration (x-axis). The experimental details are described in Methods. The CDR sequences of these IgGs are shown in Supplementary Table S3, and the numerical values of the IC_{50}'s are listed as >10^5 ng/mL in Fig. 8 and Supplementary Table S4. The error bars associated with the data points are calculated with at least three independent repeats of the microneutralization assay.

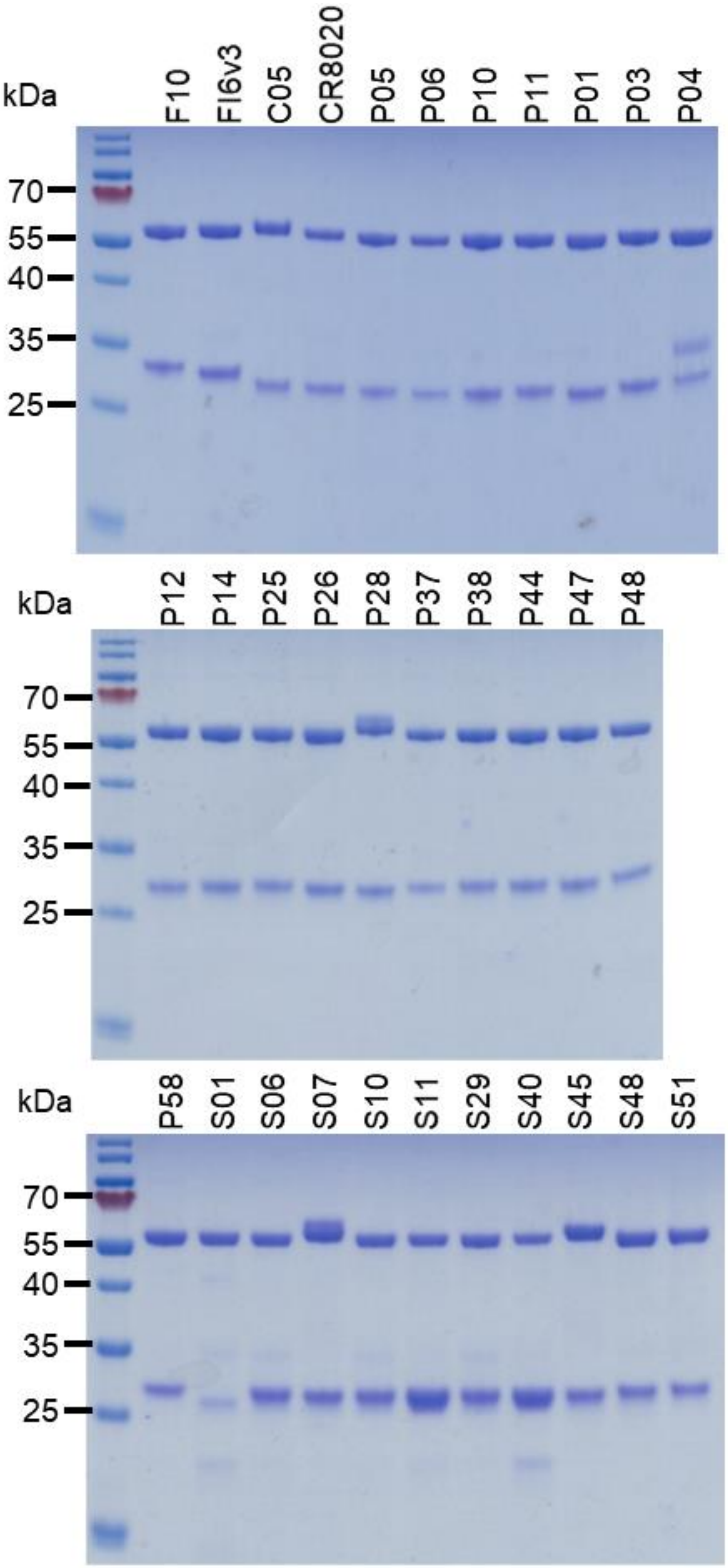

Supplementary Figure S3. SDS-PAGE analysis of purified IgGs. Two microgram of purified IgGs were analyzed by the SDS-PAGE under reducing condition. P04 is glycosylated in the CDR-L1, and P28, S07 and S45 are glycosylated in the CDR-H1.

Supplementary Table S1. Primers for diversifying CDR-L1, L2, L3, H1, H2 in GH2-5~24 antibody libraries [2].

Target CDRs	Primer	Sequences[a]	Mutagenized residues[b]									
		GACCATTACCTGCCGTGCGAGCCAGGATG TT AGC ACG GCG GTCGCATGGTATCAGCAGAAACCA	S30	T31	A32							
	L101	GACCATTACCTGCCGTGCGAGCCAGGATG TT THY THY THY GTCGCATGGTATCAGCAGAAACCA	b	b	b							
	L102	GACCATTACCTGCCGTGCGAGCCAGGATG TT THY THY KGG GTCGCATGGTATCAGCAGAAACCA	b	b	a							
	L103	GACCATTACCTGCCGTGCGAGCCAGGATG TT THY KGG KGG GTCGCATGGTATCAGCAGAAACCA	b	a	a							
	L104	GACCATTACCTGCCGTGCGAGCCAGGATG TT THY KGG THY GTCGCATGGTATCAGCAGAAACCA	b	a	b							
	L105	GACCATTACCTGCCGTGCGAGCCAGGATG TT KGG THY THY GTCGCATGGTATCAGCAGAAACCA	a	b	b							
	L106	GACCATTACCTGCCGTGCGAGCCAGGATG TT KGG THY KGG GTCGCATGGTATCAGCAGAAACCA	a	b	a							
	L107	GACCATTACCTGCCGTGCGAGCCAGGATG TT KGG KGG KGG GTCGCATGGTATCAGCAGAAACCA	a	a	a							
	L108	GACCATTACCTGCCGTGCGAGCCAGGATG TT KGG KGG THY GTCGCATGGTATCAGCAGAAACCA	a	a	b							
	L109	GACCATTACCTGCCGTGCGAGCCAGGATG TT RRY RRY RRY GTCGCATGGTATCAGCAGAAACCA	d	d	d							
		GGCAAAGCGCCGAAACTTCTGATA TAC TCT GCG TCC TTC CTGTATAGCGGCGTGCCGTCGCGTTTTTCG	Y49	S50	A51	S52	F53					
	L201	GGCAAAGCGCCGAAACTTCTGATA THY THY NCN VSY THY CTGTATAGCGGCGTGCCGTCGCGTTTTTCG	b	b	p	e	b					
	L202	GGCAAAGCGCCGAAACTTCTGATA THY THY NCN VSY KGG CTGTATAGCGGCGTGCCGTCGCGTTTTTCG	b	b	p	e	a					
	L203	GGCAAAGCGCCGAAACTTCTGATA THY KGG NCN VSY KGG CTGTATAGCGGCGTGCCGTCGCGTTTTTCG	b	a	p	e	a					
	L204	GGCAAAGCGCCGAAACTTCTGATA THY KGG NCN VSY KGG CTGTATAGCGGCGTGCCGTCGCGTTTTTCG	b	b	p	e	b					
		GATTTTGCGACCTACTACTGTCAACAG CAT TAT ACC ACA CCG CCG ACCTTCGGTCAAGGCACCAAAGTGG	H91	Y92	T93	T94	P95	P96				

	L301	GATTTTGCGACCTACTACTGTCAACAG THY THY RRY THY CCG NTN ACCTTCGGTCAAGGCACCAAAGTGG	b	b	d	b	P	z				
	L302	GATTTTGCGACCTACTACTGTCAACAG THY THY RRY KGG CCG NTN ACCTTCGGTCAAGGCACCAAAGTGG	b	b	d	a	P	z				
	L303	GATTTTGCGACCTACTACTGTCAACAG THY KGG RRY KGG CCG NTN ACCTTCGGTCAAGGCACCAAAGTGG	b	a	d	a	P	z				
	L304	GATTTTGCGACCTACTACTGTCAACAG THY KGG RRY THY CCG NTN ACCTTCGGTCAAGGCACCAAAGTGG	b	a	d	b	P	z				
	L305	GATTTTGCGACCTACTACTGTCAACAG KGG THY RRY THY CCG NTN ACCTTCGGTCAAGGCACCAAAGTGG	a	b	d	b	P	z				
	L306	GATTTTGCGACCTACTACTGTCAACAG KGG THY RRY KGG CCG NTN ACCTTCGGTCAAGGCACCAAAGTGG	a	b	d	a	P	z				
	L307	GATTTTGCGACCTACTACTGTCAACAG KGG KGG RRY KGG CCG NTN ACCTTCGGTCAAGGCACCAAAGTGG	a	a	d	a	P	z				
	L308	GATTTTGCGACCTACTACTGTCAACAG KGG KGG RRY THY CCG NTN ACCTTCGGTCAAGGCACCAAAGTGG	a	a	d	b	P	z				
		GAGCTGTGCGGCGAGCGGGTTCACCATT AGC GAT TAC TGG ATTCATTGGGTGCGTCAAGCTCCCG	S30	D31	Y32	W33						
	H101	GAGCTGTGCGGCGAGCGGGTTCACCATT RRY RRY THY THY ATTCATTGGGTGCGTCAAGCTCCCG	d	d	b	b						
	H102	GAGCTGTGCGGCGAGCGGGTTCACCATT RRY RRY THY KGG ATTCATTGGGTGCGTCAAGCTCCCG	d	d	b	a						
	H103	GAGCTGTGCGGCGAGCGGGTTCACCATT RRY RRY KGG THY ATTCATTGGGTGCGTCAAGCTCCCG	d	d	a	b						
	H104	GAGCTGTGCGGCGAGCGGGTTCACCATT RRY RRY KGG KGG ATTCATTGGGTGCGTCAAGCTCCCG	d	d	a	a						
		GCAAGGGGCTGGAGTGGGTCGCG GGC ATT ACG CCC GCT GGC GGT TAC ACA TAT TATGCCGACAGCGTGAAAGGTCGCTTTACGA	G50	I51	T52	P52A	A53	G54	G55	Y56	T57	Y58
	H201	GCAAGGGGCTGGAGTGGGTCGCG KGG ATT KGG CCC THY KGG GGT THY ACA THY TATGCCGACAGCGTGAAAGGTCGCTTTACGA	a	I	a	P	b	a	G	b	T	b
	H202	GCAAGGGGCTGGAGTGGGTCGCG THY ATT KGG CCC THY KGG GGT THY ACA THY TATGCCGACAGCGTGAAAGGTCGCTTTACGA	b	I	a	P	b	a	G	b	T	b

[a] Codons for mutagenized residues at CDR regions are underlined; DNA degeneracies are represented by IUB code (N = A/T/G/C, H = A/C/T, V = A/C/G, K = G/T, R = A/G, S = G/C, W = A/T, and Y = C/T).

[b] Residues are in Kabat number. Symbols used: a, W/G; b, F/S/Y; d, G/D/S/N; e, G/A/S/T/R/P; p, A/T/P/S; q, F/Y/D/V/N/I/H/L; z, L/I/V/F/M.

Supplementary Table S2. Summary of CDR-H3 designs for GH2-5~24 phage-displayed synthetic scFv libraries.

Library name	Sequence designs
GH2-5	AwayY<br>AwbyY
GH2-6	AwabyY<br>AwbayY
GH2-7	AwabbyY<br>AwbabyY<br>AwbbayY
GH2-8	AwababyY<br>AwabbayY<br>AwbabayY
GH2-9	ARabbabDY<br>ARabbbaDY<br>ARbabbaDY
GH2-10	ARabbabbDY<br>ARabbbabDY<br>ARabbbbaDY<br>ARbabbabDY<br>ARbabbbaDY<br>ARbbabbaDY
GH2-11	ARabbabbbDY<br>ARabbbabbDY<br>ARabbbbabDY<br>ARabbbbbaDY<br>ARbabbabbDY<br>ARbabbbabDY<br>ARbabbbbaDY<br>ARbbabbabDY<br>ARbbabbbaDY<br>ARbbbabbaDY
GH2-12	ARFqsttsqMDY
GH2-13	ARFqqqqqaaMDY<br>ARFqqqqaqaMDY<br>ARFqqqaqqaMDY

	ARFqqaqqqaMDY<br>ARFqaqqqqaMDY<br>ARFaqqqqqaMDY<br>ARFqqqqaaqMDY<br>ARFqqqaqaqMDY<br>ARFqqaqqaqMDY<br>ARFqaqqqaqMDY<br>ARFaqqqqaqMDY<br>ARFqqqaaqqMDY<br>ARFqqaqaqqMDY<br>ARFqaqqaqqMDY<br>ARFaqqqaqqMDY<br>ARFqqaaqqqMDY<br>ARFqaqaqqqMDY<br>ARFaqqaqqqMDY<br>ARFqaaqqqqMDY<br>ARFaqaqqqqMDY<br>ARFaaqqqqqMDY
GH2-14	ARFqststtsqMDY<br>ARFqsttstsqMDY
GH2-16	ARFqsysttsysqMDY<br>ARFqsytsttysqMDY<br>ARFqsyttstysqMDY
GH2-18	ARFqsystttt sysqMDY<br>ARFqsyysttsyysqMDY<br>ARFqsyytsttyysqMDY<br>ARFqsyyttstyysqMDY
GH2-20	ARFqsysytttt ysysqMDY<br>ARFqsyystttt syysqMDY<br>ARFqsyyysttsyyysqMDY<br>ARFqsyyytsttyyysqMDY<br>ARFqsyyyttstyyysqMDY
GH2-22	ARFqsysyytttt yysysqMDY<br>ARFqsyysytttt ysyysqMDY<br>ARFqsyyystttt syyysqMDY<br>ARFqsyyyysttsyyyysqMDY<br>ARFqsyyyytsttyyyysqMDY

	**ARFqsyyyyttstyyyysqMDY**
GH2-24	**ARFqsysyyyttttyyysysqMDY**<br>**ARFqsyysyyttttyysyysqMDY**<br>**ARFqsyyysyttttysyyysqMDY**<br>**ARFqsyyyysttttsyyyysqMDY**<br>**ARFqsyyyyysttsyyyyysqMDY**<br>**ARFqsyyyyytsttyyyyysqMDY**<br>**ARFqsyyyyyttstyyyyysqMDY**
	Encoded amino acid types
a	WG
b	FSY
q	FYDVNIHL
w	RGW
y	NDYH
s	GS
t	YS

Supplementary Table S3. CDR sequences of anti-influenza IgGs derived from synthetic antibody libraries

Antibody	CDR L1	CDR L2	CDR L3	CDR H1	CDR H2	CDR H3	Library
F10	TGNSNNVGNQGAA	YRNNDRPS	STWDSSLSAV	TSSEVTFSSFAIS	GISPMFGTPN	ARSPSYICSGGTCVFDH	
FI6V3	KSSQSVTFNYKNYLA	YWASTRES	QQHYRTPP	AASGFTFSTYAMH	VISYDANYKY	AKDSQLRSLLYFEWLSQGYFDY	
C05	QASQDIRKFLN	YDASNLQR	QQYDGLPF	VGSGSSFGESTLSY YAVS	IINAGGGDID	AKHMSMQQVVSAGWERADLV GDAFDV	
CR8020	RASQSVSMNYLA	YGASRRAT	QQYGTSPR	KASGYTFTSFGVS	WISAYNGDTY	AREPPLFYSSWSLDN	
Do1	RASQDVWGGVA	FFSRYLYS	QQYYNGPL	AASGFTIDNGSIH	WIGPYGGFTS	ARFYGSGSSSFMDY	GH2-14
P01	RASQDVGWYVA	YGSTFLYS	QQYYDSPL	AASGFTIGDGSIH	WIGPYGGSTF	ARFFWGINMDY	GH2-11
P03	RASQDVWGYVA	SWSGSLYS	QQYYNWPV	AASGFTIDSFGIH	FIGPFGGSTF	ARFDSNSYSYHGIMDY	GH2-16
P04	RASQDVNGSVA	SSSASLYS	QQGWSYPL	AASGFTINSWSIH	SIWPFGGFTF	ARGYSSFGDY	GH2-10
P05	RASQDVGSSVA	SSSPYLYS	QQYYDYPL	AASGFTINDYGIH	GIWPYWGFTF	ARFHGSSYYSVMDY	GH2-14
P06	RASQDVGGGVA	SGTSGLYS	QQSSNFPI	AASGFTIGGYWIH	GIGPYWGSTY	ARFNNWFWNVMDY	GH2-13
P10	RASQDVNSNVA	YWAGYLYS	QQSSDFPI	AASGFTIDNSWIH	SIWPFGGYTY	ARFGNVFDWYMDY	GH2-13
P11	RASQDVDNNVA	SYASWLYS	QQSSGGPV	AASGFTISSFWIH	GIGPFWGSTF	ARFNDWFYHGMDY	GH2-13
P12	RASQDVGYWVA	YWTSGLYS	QQYSNWPI	AASGFTIGDYYIH	GIGPSWGSTS	ARFYNNHWGFMDY	GH2-13
P14	RASQDVGFYVA	SWSSYLYS	QQYYNYPL	AASGFTIGDFGIH	GIWPFGGYTY	ARFVNWDGDYMDY	GH2-13
P25	RASQDVWGYVA	SGSRSLYS	QQYYNYPI	AASGFTISNGGIH	GIGPYGGYTY	ARFYGYSGIMDY	GH2-12
P26	RASQDVWSGVA	YGTTYLYS	QQYYSFLL	AASGFTIDNSWIH	SIGPYWGYTS	ARFVFFLPYAMDY	GH2-13
P28	RASQDVWYSVA	YFATGLYS	QQYFNWPV	AASGFTINNSGIH	SIWPSGGYTY	ARFNSSYSSGLMDY	GH2-14
P37	RASQDVGNDVA	SSARGLYS	QQYYNFPI	AASGFTFNSWGIH	GIWPYWGFTY	ARFHGSSYYSVMDY	GH2-14
P38	RASQDVWGYVA	SWPGGLYS	QQYSSFPL	AASGFTINDGGIH	FIGPYGGSTF	AGFIGDYSSYHGVMDY	GH2-16
P44	RASQDVNNNVA	YWSSSLYS	QQYYNFPV	AASGFTIDGWWIH	GIWPFGGFTS	ARSYSGYSGDY	GH2-11
P47	RASQDVYYYVA	SGSSYLYS	QQYYNWPL	AASGFTIGNSGIH	SIWPSGGSTY	ARFGHIDGDIMDY	GH2-13
P48	RASQDVWSYVA	SYTSYLYS	QQYFNWPI	AASGFTINSWGIH	GIGPSWGYTS	ARFGDGDFDLMDY	GH2-13
P58	RASQDVYSYVA	SSSRGLYS	QQYSSFPI	AASGFTIGGGGIH	WIWPYWGYTY	ARFNSYSYSGVMDY	GH2-14
S01	RASQDVWGYVA	SFPSSLYS	QQYYDGPV	AASGFTINNYGIH	SIWPSGGYTS	ARFGLGDYDIMDY	GH2-13
S06	RASQDVSSWVA	YGTTFLHS	QQYYNGPL	AASGFTIGGGWIH	FIGPYGGSTF	ARFNFGFWNHMDY	GH2-13
S07	RASQDVWGYVA	SYASFLYS	QQYFNWPV	AASGFTINSSGIH	SIGPSWGSTY	ARFGIGDIDVMDY	GH2-13
S10	RASQDVSYYVA	FWSTFLYS	QQYYDSPM	AASGFTIGGYGIH	SIGPSWGFTF	ARFNWVINGVTDY	GH2-13
S11	RASQDVYSWVA	YYSSFLYS	QQYYNGPL	AASGFTIDNGGIH	WIGPYGGSTS	ARFGFGLHDLMDY	GH2-13
S29	RASQDVFGGVA	YWSSWLYS	QQYYDGPI	AASGFTISDYWIH	SIWPSGGYTY	ARFNWIVGHYMDY	GH2-13
S40	RASQDVGFYVA	SWSSYLYS	QQYYNYPL	AASGFTIGDFGIH	GIWPFGGYTY	ARFVNWDGDYMDY	GH2-13
S45	RASQDVGWWVA	YGARFLYS	QQYFNGPL	AASGFTINGSSIH	YIGPFGGSTY	ARFWHGYNLYMDY	GH2-13
S48	RASQDVGWWVA	YGTRWLYS	QQYYSGPI	AVSGFTIGDGSIH	SIGPYGGSTY	ARFHYGYWNNMDY	GH2-13
S51	RASQDVWGYVA	SYTTYLYS	QQYYNSPV	AASGFTIDDWGIH	WIWPYGGFTS	ARFGFVDWNLMDY	GH2-13

Supplementary Table S4. Protein expression, epitope grouping and assessment of the neutralizing and binding potencies of anti-influenza IgGs derived from synthetic antibody libraries.

Name	Yield (mg/L)	Epitope Grouping	EC_{50} of ELISA binding (EC_{50} (ng/ml) (correlation coefficient))				EC_{50} of cell surface binding (EC_{50} (ng/ml) (max. MFI))			IC_{50} of true virus neutralization (ng/ml)	IC_{50} of pseudo virus neutralization (ng/ml)			
			H1N1 CA/09 HA	H3N2 WN/05 HA	H5N1 VN/04 HA	H7N9 AH/13 HA	H1N1 CA/09 HA	H3N2 WN/05 HA	H5N1 VN/04 HA	H1N1 CA/09	H1N1 CA/09	H3N2 WN/05	H5N1 VN/04	H7N9 AH/13
F10	5.68	I	12.15 (0.96)	NB	20.98 (0.94)	NB	1051 (9527)	NB (220)	590 (10535)	1256.98	11.9	ND	12	$>2\times10^5$
FI6V3	9.87	I	10.68 (0.96)	78.93 (0.92)	6.48 (0.92)	313.63 (0.95)	67 (10125)	65 (16583)	610 (13712)	9214.55	21.3	ND	13	51
C05	3.48		NB	8.44 (0.95)	NB	NB	NB (86)	130 (14588)	NB (547)	$>5\times10^5$	$>10^5$	10.17	$>2\times10^5$	$>2\times10^5$
CR8020	1.80		NB	19.37 (0.97)	NB	68.99 (0.92)	NB (91)	569 (18011)	NB (639)	$>5\times10^5$	$>10^5$	47.66	$>2\times10^5$	52
Do1	9.15		NB	NB	$>10^4$	NB	NB (228)	NB (278)	NB (313)	$>5\times10^5$	$>10^5$	$>2\times10^4$	$>2\times10^5$	$>2\times10^5$
P01	12.60	II	54.47 (0.94)	NB	185.09 (0.96)	NB	5900 (3668)	NB (342)	5940 (1195)	$>5\times10^5$	$>10^5$	$>2\times10^4$	$>2\times10^5$	$>2\times10^5$
P03	13.23	III	15.61 (0.96)	$>10^4$	$>10^4$	NB	494 (6022)	NB (147)	NB (216)	$>5\times10^5$	$>10^5$	$>2\times10^4$	$>2\times10^5$	$>2\times10^5$
P04	9.50	I	13.72 (0.97)	NB	>2000	NB	1920 (6244)	3500 (2147)	3260 (1603)	62323	146.3	$>2\times10^4$	185	$>2\times10^5$
P05	15.00	I	16.50 (0.97)	NB	NB	NB	2060 (7950)	NB (176)	190 (1723)	289733	36.6	$>2\times10^4$	$>2\times10^5$	$>2\times10^5$
P06	18.00	I	5.59 (0.92)	NB	238.05 (0.98)	NB	1560 (11619)	NB (250)	260 (1356)	135818	135.9	$>2\times10^4$	1256	$>2\times10^5$
P10	4.93	I	17.40 (0.97)	NB	NB	NB	410 (9663)	NB (117)	NB (517)	$>5\times10^5$	464.9	$>2\times10^4$	$>2\times10^5$	$>2\times10^5$
P11	23.40	I	29.22 (0.95)	NB	$>10^4$	NB	300 (8164)	NB (303)	2420 (924)	953155	104	$>2\times10^4$	9293.78	$>2\times10^5$
P12	12.38	II	54.94 (0.94)	$>10^4$	NB	NB	5860 (1995)	NB (209)	6540 (883)	$>5\times10^5$	$>10^5$	$>2\times10^4$	$>2\times10^5$	$>2\times10^5$
P14	17.43	III	11.71 (0.96)	NB	NB	NB	125 (8337)	NB (154)	NB (328)	4393351	$>10^5$	$>2\times10^4$	$>2\times10^5$	$>2\times10^5$
P25	6.13	III	138.93 (0.95)	NB	NB	NB	761 (2376)	NB (152)	NB (318)	$>5\times10^5$	$>10^5$	$>2\times10^4$	$>2\times10^5$	$>2\times10^5$
P26	5.45	I	11.91 (0.96)	NB	26.55 (0.92)	75.05 (0.94)	2035 (8923)	NB (120)	650 (6402)	29596	549.3	$>2\times10^4$	85	$>2\times10^5$
P28	13.70	II	797.79 (0.91)	NB	NB	NB	NB (168)	NB (127)	NB (193)	$>5\times10^5$	$>10^5$	$>2\times10^4$	$>2\times10^5$	$>2\times10^5$
P37	22.00	I	235.68 (0.98)	NB	NB	NB	420 (5028)	NB (199)	1180 (644)	$>5\times10^5$	1674	$>2\times10^4$	$>2\times10^5$	$>2\times10^5$
P38	22.40	III	94.39 (0.96)	NB	NB	NB	3160 (988)	NB (137)	NB (240)	$>5\times10^5$	$>10^5$	$>2\times10^4$	$>2\times10^5$	$>2\times10^5$
P44	9.50	II	15.03 (0.97)	NB	100 (0.96)	162.86 (0.94)	466 (8478)	NB (127)	NB (188)	$>5\times10^5$	$>10^5$	$>2\times10^4$	$>2\times10^5$	$>2\times10^5$
P47	6.93	III	9.62 (0.96)	NB	NB	NB	610 (10241)	NB (175)	NB (396)	$>5\times10^5$	$>10^5$	$>2\times10^4$	$>2\times10^5$	$>2\times10^5$
P48	2.80	III	15.36 (0.97)	NB	NB	NB	2400 (7865)	NB (258)	NB (474)	$>5\times10^5$	$>10^5$	$>2\times10^4$	$>2\times10^5$	$>2\times10^5$

**P58**	**0.90**	**II**	**22.74 (0.96)**	**NB**	**$>10^4$**	**503.58 (0.93)**	**250 (6288)**	**3581 (697)**	**366 (6713)**	**$>5\times10^5$**	**$>10^5$**	**$>2\times10^4$**	**$>2\times10^5$**	**$>2\times10^5$**
**S01**	**9.65**	**III**	**12.18 (0.96)**	**NB**	**NB**	**NB**	**384 (8428)**	**NB (178)**	**NB (306)**	**$>5\times10^5$**	**$>10^5$**	**$>2\times10^4$**	**$>2\times10^5$**	**$>2\times10^5$**
**S06**	**9.48**	**II**	**46.70 (0.97)**	**NB**	**310.98 (0.97)**	**NB**	**3982 (1648)**	**NB (135)**	**NB (249)**	**$>5\times10^5$**	**$>10^5$**	**$>2\times10^4$**	**$>2\times10^5$**	**$>2\times10^5$**
**S07**	**16.43**	**III**	**11.42 (0.96)**	**NB**	**NB**	**NB**	**1610 (10791)**	**3710 (410)**	**4980 (863)**	**$>5\times10^5$**	**$>10^5$**	**$>2\times10^4$**	**$>2\times10^5$**	**$>2\times10^5$**
**S10**	**3.45**	**I**	**14.00 (0.97)**	**NB**	**259.21 (0.97)**	**NB**	**3380 (10696)**	**7860 (511)**	**790 (1099)**	**1562813**	**349.1**	**$>2\times10^4$**	**267**	**$>2\times10^5$**
**S11**	**16.98**	**II**	**26.79 (0.98)**	**NB**	**78.14 (0.90)**	**NB**	**4070 (5600)**	**NB (207)**	**NB (434)**	**$>5\times10^5$**	**$>10^5$**	**$>2\times10^4$**	**$>2\times10^5$**	**$>2\times10^5$**
**S29**	**18.18**	**I**	**22.27 (0.98)**	**NB**	**$>10^4$**	**NB**	**4220 (6276)**	**NB (188)**	**NB (367)**	**$>5\times10^5$**	**672.2**	**$>2\times10^4$**	**$>2\times10^5$**	**$>2\times10^5$**
**S40**	**24.05**	**III**	**16.32 (0.96)**	**NB**	**NB**	**NB**	**460 (8626)**	**NB (109)**	**2850 (724)**	**435924**	**$>10^5$**	**$>2\times10^4$**	**$>2\times10^5$**	**$>2\times10^5$**
**S45**	**16.68**	**II**	**20.61 (0.96)**	**NB**	**230.35 (0.97)**	**NB**	**4470 (2473)**	**15800 (529)**	**NB (451)**	**$>5\times10^5$**	**$>10^5$**	**$>2\times10^4$**	**$>2\times10^5$**	**$>2\times10^5$**
**S48**	**10.03**	**II**	**12.50 (0.97)**	**NB**	**73.75 (0.91)**	**NB**	**4860 (6243)**	**NB (122)**	**1920 (875)**	**$>5\times10^5$**	**$>10^5$**	**$>2\times10^4$**	**$>2\times10^5$**	**$>2\times10^5$**
**S51**	**6.75**	**III**	**23.38 (0.96)**	**NB**	**NB**	**NB**	**754 (6290)**	**NB (340)**	**NB (452)**	**$>5\times10^5$**	**$>10^5$**	**$>2\times10^4$**	**$>2\times10^5$**	**$>2\times10^5$**

Supplementary Table S5. Crystallography parameters for the S40-HA complex structure

	**HA1/S40-Fab**
**Data collection**	
Wavelength (Å)	0.9
Space group	*C*2
Cell dimensions (Å)	a=200.40, b=133.64, c=133.14, α=90, β=110.47,
Resolution (Å)	25.0-3.35 (3.47-3.35)
Unique reflections	47,477
R_{merge} (%)	9.6 (64.5)
$I/\sigma(I)$	17.3 (2.4)
Completeness	99.6 (100.0)
Redundancy	4.6 (4.7)
**Refinement**	
Resolution (Å)	25.0-3.35
No. of reflections R_{work}/R_{free}	34,487/1,826
R_{work}/R_{free}	23.0/27.5
No. of protein atoms/Avg B factor (Å^2)	11,100/176.1
**RMSD**	
Bond lengths (Å) /Bond angles (°)	0.01/1.49
**Ramachandran statistics (%)[b]**	
Most favored	75.0
Additionally allowed	22.8
Generously allowed	1.6
Disallowed	0.6

[a] Values corresponding to the highest resolution shells are shown in parentheses.

[b] Stereochemistry of the model was validated with PROCHECK.

Reference:

1 Sidhu, S. S. & Weiss, G. A. in *Phage Display* (eds T. Clackson & H. B. Lowman) 27-41 (Oxford University Press, 2004).

2 Chen, H. S. *et al.* Predominant structural configuration of natural antibody repertoires enables potent antibody responses against protein antigens. *Scientific reports* **5**, 12411, doi:10.1038/srep12411 (2015).

3 Andris-Widhopf, J., Steinberger, P., Fuller, R., Rader, C. & Barbas, C. F., 3rd. Generation of human scFv antibody libraries: PCR amplification and assembly of light- and heavy-chain coding sequences. *Cold Spring Harb Protoc* **2011**, doi:2011/9/pdb.prot065573 [pii]
10.1101/pdb.prot065573 [doi] (2011).

4 Yu, C.-M. *et al.* Rationalization and Design of the Complementarity Determining Region Sequences in an Antibody-Antigen Recognition Interface. *PLoS ONE* **7**, e33340, doi:10.1371/journal.pone.0033340 (2012).
